# MURF2B, a Novel LC3-Binding Protein, Participates with MURF2A in the Switch between Autophagy and Ubiquitin Proteasome System during Differentiation of C2C12 Muscle Cells

**DOI:** 10.1371/journal.pone.0076140

**Published:** 2013-10-04

**Authors:** Véronique Pizon, Sofia Rybina, Fabien Gerbal, Florence Delort, Patrick Vicart, Giuseppe Baldacci, Eric Karsenti

**Affiliations:** 1 University Paris Diderot, Sorbonne Paris Cité, Unité de Biologie Fonctionnelle et Adaptative, CNRS EAC4413, Paris, France; 2 European Molecular Biology Laboratory, Heidelberg, Germany; 3 Université Paris Diderot, Matière et Systèmes Complexes, CNRS UMR 7057, Paris, France; 4 Université Paris Diderot, CNRS, Institut Jacques Monod, Paris, France; 5 Université Pierre et Marie Curie, Physics Department-UFR925, Paris, France; IISER-TVM, India

## Abstract

The ubiquitin proteasome system and macroautophagy are proteolytic pathways essential in the maintenance of cellular homeostasis during differentiation and remodelling of skeletal muscle. In both pathways, proteins to be degraded are tagged with polyubiquitin. In skeletal muscles, the MURF2 proteins display E3 ubiquitin ligase structure suggesting that they may covalently attach ubiquitin polypeptides to still unknown target proteins. So far only MURF2A isoforms were studied and shown to interact with p62/SQSTM1, a protein implicated in macroautophagic and ubiquitin proteasome system degradations. Here, we analyzed the MURF2B and MURF2A proteins and show that the ratio of the isoforms changes during differentiation of muscle C2C12 cells and that the shift of the isoforms expression follows the sequential activation of autophagic or proteasomal degradation. We also show that MURF2B has a functional domain needed for its interaction with LC3, a protein needed for autophagic vesicles formation. Using specific MURF2 RNAi cells we observed that MURF2A and MURF2B are both needed for the formation of autophagosomes and that in the absence of MURF2B, the cells expressing MURF2A display an activated ubiquitin proteasome system implicated in the degradation of p62/SQSTM1 by UPS. Altogether, our results indicate that MURF2A and MURF2B proteins could participate in the molecular switch between the two ubiquitin degradative pathways.

## Introduction

**Figure 1:**
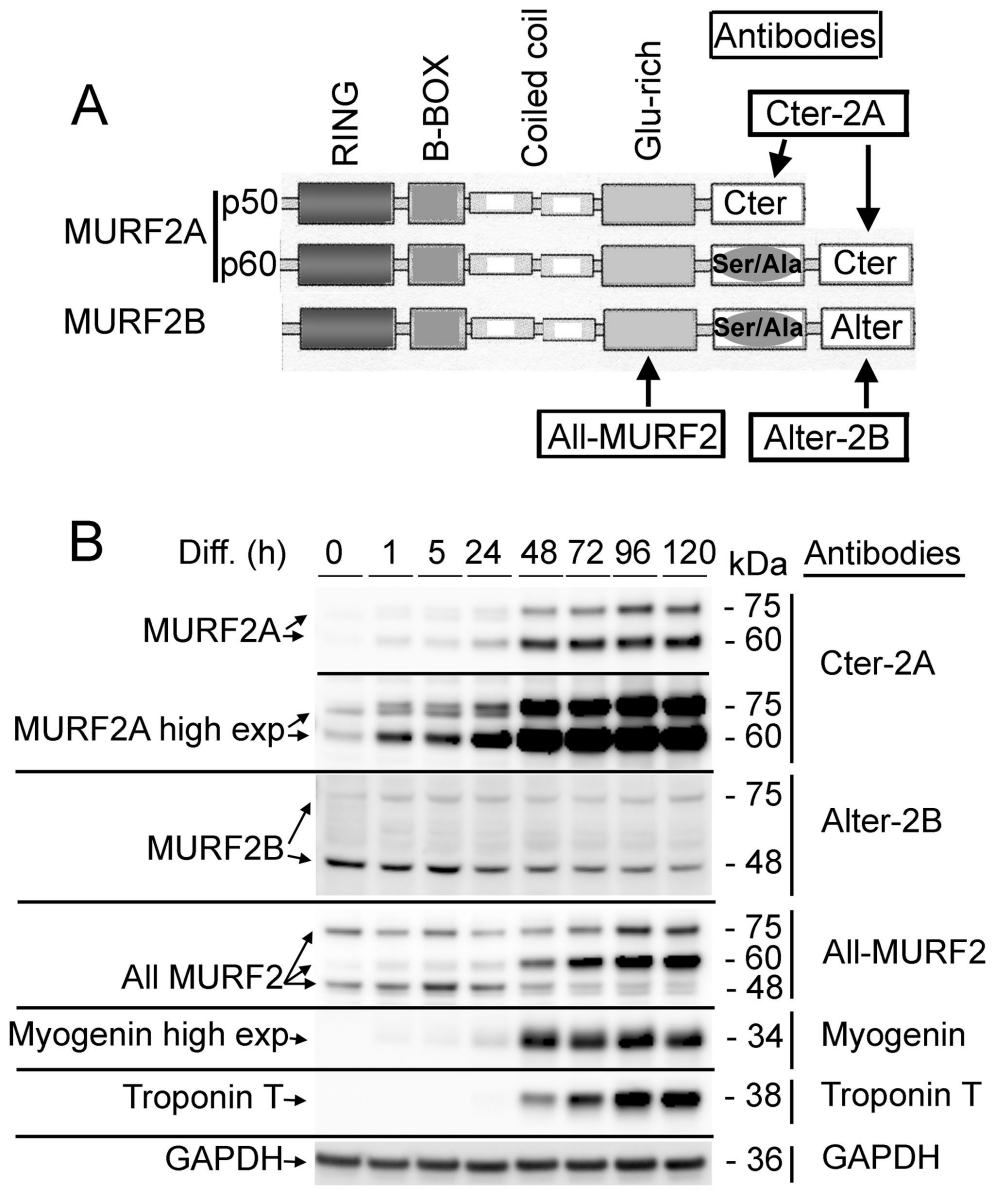
MURF2 expression during differentiation of C2C12 cells. (A) Schematic structure of the MURF2 isoforms of skeletal muscle cells and locations of the epitopes recognized by various antibodies (names are framed). (B) Same amounts of C2C12 lysates obtained at various time of differentiation (Diff.) were used for Western blots analyses and probed with the indicated antibodies.

During muscle differentiation and muscle activity, proteins undergo continuous turnover tightly regulated by hormones and nutrients to maintain functional sarcomeres. During this process, syntheses and degradations must be accurately orchestrated to preserve muscle integrity. Two major degradation pathways are implicated in protein quality control of the sarcomere: macroautophagy (hereafter referred as autophagy) and the ubiquitin proteasome system (UPS) [1,2,3]. In muscles, the crosstalk between autophagy and UPS is mainly regulated by the Akt-FOXO3 regulatory pathway [4]. Autophagy is a lysosomal degradative process that begins with the formation of pre-autophagosomal sequestering cisterns that subsequently give rise to crescent-shaped isolation membranes or phagophores [5]. Phagophores elongate and expand around a portion of cytoplasm to eventually close upon themselves to form double-membrane vesicles, the autophagosomes. When autophagosomes are completed, they fuse with lysosomes to form the autolysosomes where degradations occur. Numerous proteins are involved in these processes. Vesicles nucleation needs the recruitment of Atg proteins to the phagophore assembly site and the activation of a phosphatidylinositol 3-kinase (PtdIns3K) complex [6]. During vesicles expansion and completion, LC3, the mammalian homolog of Atg8, is processed by the Atg4 protease to expose its C-terminal glycine giving LC3-I, LC3-I is then conjugated to phosphatidylethanolamine (PE) [7]. This conjugation is fundamental for autophagosome formation and the conjugated form LC3-II, associated tightly with the external membrane of autophagosomes and autolysosomes, serves as autophagic marker [8]. Two major proteins also implicated in autophagy are p62/SQSTM1 (hereafter named p62) and its interacting partner NBR1. P62 and NBR1 act as cargo receptors for degradation of ubiquitinated substrates and are themselves degraded by autophagy. To achieve autophagy, p62 and NBR1 proteins must interact with LC3 via their LC3-interacting sequences also named the LIR domain [9,10,11]. UPS is a large non-lysosomal degradative complex composed of the 20S proteasome associated with a 19S regulatory complex to form the 26S proteasome in which ubiquitinated substrates are target then degraded [12,13,14]. Chains of four and more ubiquitin moieties mark proteins for degradation by the 26S proteasome. Ubiquitination is catalyzed by a cascade of three enzymes. The ubiquitin-activating enzyme (E1) activates ubiquitin, which is then transferred to a ubiquitin carrier protein (E2). Then E2 interacts with E3, an ubiquitin ligase, to catalyze transfer of ubiquitin to a protein substrate. After recognition and unfolding, the substrate can be degraded by one of the 20S multicatalytic protease activities. Among the various E3 ligases identified in striated skeletal muscles, MURF1, MURF2 and MURF3 proteins are highly homologous and can homo- and heterodimerize [15]. Differential splicing in the MURF3 and MURF2 genes, produces various isoforms specifically expressed according to the muscle fiber type [16]. Three MURF2 protein isoforms were identified [17,18]. Two different types of C-terminus domains exist in the MURF2 isoforms (Figure 1A): the A-type sequence found in one 50 kDa and one 60 kDa protein designated respectively MURF2A p50 and MURF2A p60 (both isoforms are referred to MURF2A and their C-terminus as Cter) and the B-type sequence only found in a 60 kDa protein (hereafter referred to MURF2B with an Alternative C-terminus named Alter). All members of the MURF2 protein family display a common N-terminus domain containing a RING Zinc-finger and a B-box Zinc finger domain, hallmarks of the E3 ubiquitin ligase proteins engaged in UPS degradation. So far only the MURF2A isoforms were studied since no antibodies were available to detect the MURF2B protein. MURF2A isoforms direct the association of titin and myosin with microtubules (MTs) during myogenic differentiation and are involved in MTs stability [17]. The MURF2A isoforms are also implicated in a mechanotransduction pathway in which titin interacts with a complex formed with NBR1 and p62, which in turn interacts with MURF2A that ultimately ligates the serum response factor, an ubiquitous nuclear transcription factor [19]. Recently, analysis of MURF2A expression during mouse embryonic skeletal muscle development showed that expression of MURF2A p50 and MURF2A p60 isoforms are developementally regulated and parallels that of proteins involved in autophagy, namely LC3, p62 and NBR1 [16]. Altogether, these results suggested that MURF2A could participate to UPS and autophagic degradation. Since MURF2B displays 94% identity with MURF2A, we investigated if all MURF2 proteins had identical biological functions regarding degradation of ubiquitinated proteins. Therefore, we raised a specific MURF2B antibody to analyse the expression, location and biological function of MURF2A and MURF2B during differentiation of the mouse skeletal muscle C2C12 cells. We show that MURF2A and MURF2B isoforms are submitted to specific regulations and display different cellular locations in regard to p62, NBR1 and LC3 proteins. Furthermore, we discovered that MURF2B is a novel LC3-interacting protein. Using specific MURF2 RNAi cell lines we established that both isoforms are needed for autophagosome formation and that upon differentiation of muscle C2C12 cells, the ratio of endogenous MURF2B and MURF2A is unbalanced and accompanied by a transient activation of autophagy. In addition, by measuring the activity of the 20S proteasome in MURF2 RNAi and in C2C12 cells, we determined that increase of MURF2A protein paralleled the activation of UPS and could contribute to p62 degradation. Taken together, our findings indicate that the MURF2 proteins could participate to molecular switches triggering autophagic or proteasomal degradations regulating protein homeostasy.

## Results

### Expression of MURF2A and MURF2B proteins during myogenic differentiation

Among the three MURF2 isoforms expressed in striated skeletal muscle (EMBL database: accession numbers AJ243488 for MURF2Ap50, AJ243489 for MURF2Ap60 and AJ431704 for MURF2B; Figure 1A), MURF2B was never analyzed as yet. To discriminate between MURF2B and MURF2A, antibodies (designated Cter-2A for MURF2A and Alter-2B for MURF2B) were raised against the specific C-terminus of each human isoform. We also generated an antibody against a common Glu-rich domain (designated All-MURF2) to reveal all MURF2 isoforms (Figure 1A). The epitope recognized by the All-MURF2 antibody differs from the one recognized by the HP60 antibody, raised against an epitope in the Ser/Ala domain of MURF2Ap50 and MURF2Ap60 proteins [17]. The specificity of the purified Cter-2A and Alter-2B antibodies was established by the recognition of their specific epitopes tagged with GST and expressed in *E. coli* (Figure S1A) and by antibody depletion experiments (Figure S1B, C). Specificity of the purified All-MURF2 antibody was tested by Western blot analyses and by antibody depletion experiments using lysates from Hela cells expressing the full length human MURF1, MURF2B and MURF3 proteins tagged with the T7 protein (Figure S1D). These antibodies were used to examine the expression of MURF2B and MURF2A during differentiation of myogenic C2C12 cells. As previously observed [20], the Cter-2A and All-MURF2 antibodies showed that the migration of MURF2A did not follow the position expected by calculation of the molecular weights of the proteins (Figure 1B and Figure S1). Indeed, the predicted MURF2A p50 kDa and MURF2A p60 kDa isoforms migrate as proteins of 60 kDa and 75 kDa respectively. Although the predicted molecular weight of MURF2B is 60 kDa, the Alter-2B antibody revealed a major band migrating at 48 kDa and one minor band at 75 kDa. These bands were also detected with the All-MURF2 antibody, confirming that they corresponded to MURF2 proteins and could reflect unknown isoforms or post-translational modifications. When the expression of the MURF2 isoforms was analyzed during the course of differentiation in C2C12 cells, expression of MURF2B was maximal in undifferentiated myoblasts (0h) and decreased extensively after 24h of differentiation (Figure 1B). As already observed in the C57 myogenic cells [17] and in the C2C12 cells [16], MURF2A increased regularly as troponin T, a late sarcomeric marker of myogenic differentiation, upon differentiation (Figure 1B). Low amount of MURF2A protein was also detected in myoblasts by Western blot experiments after long exposure times. This expression did not result from sporadic differentiation of myoblasts since expression of myogenin, an early marker of differentiation, was not revealed even after prolonged exposure.

Collectively, these data show that MURF2A and MURF2B proteins display specific regulation during cell differentiation, maximal expression occurring in myoblasts for MURF2B and in myotubes for MURF2A.

### As a function of the ratio of their respective expression, MURF2A and MURF2B display specific location on vesicles decorated with p62, LC3 and NBR1 proteins in myoblasts.

**Figure 2:**
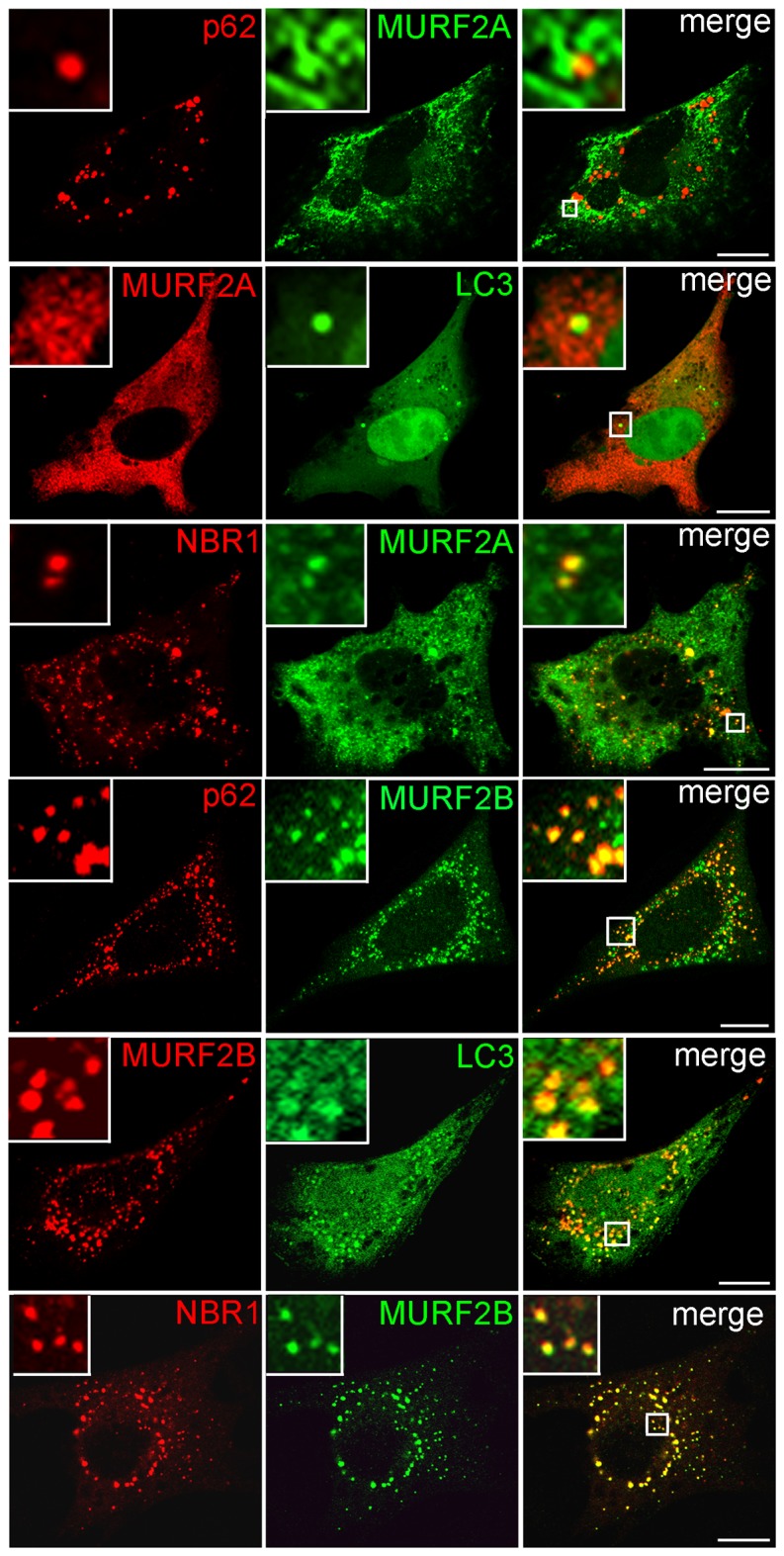
Parallel localization of MURF2 isoforms with LC3, p62 and NBR1. Plasmids expressing the indicated fluorescent proteins were used for double transfection experiments in C2C12 myoblasts. The mCherry (red) and the GFP (green) fluorescence were directly observed by confocal microscopy. Scale Bar: 10 µm.

**Figure 3:**
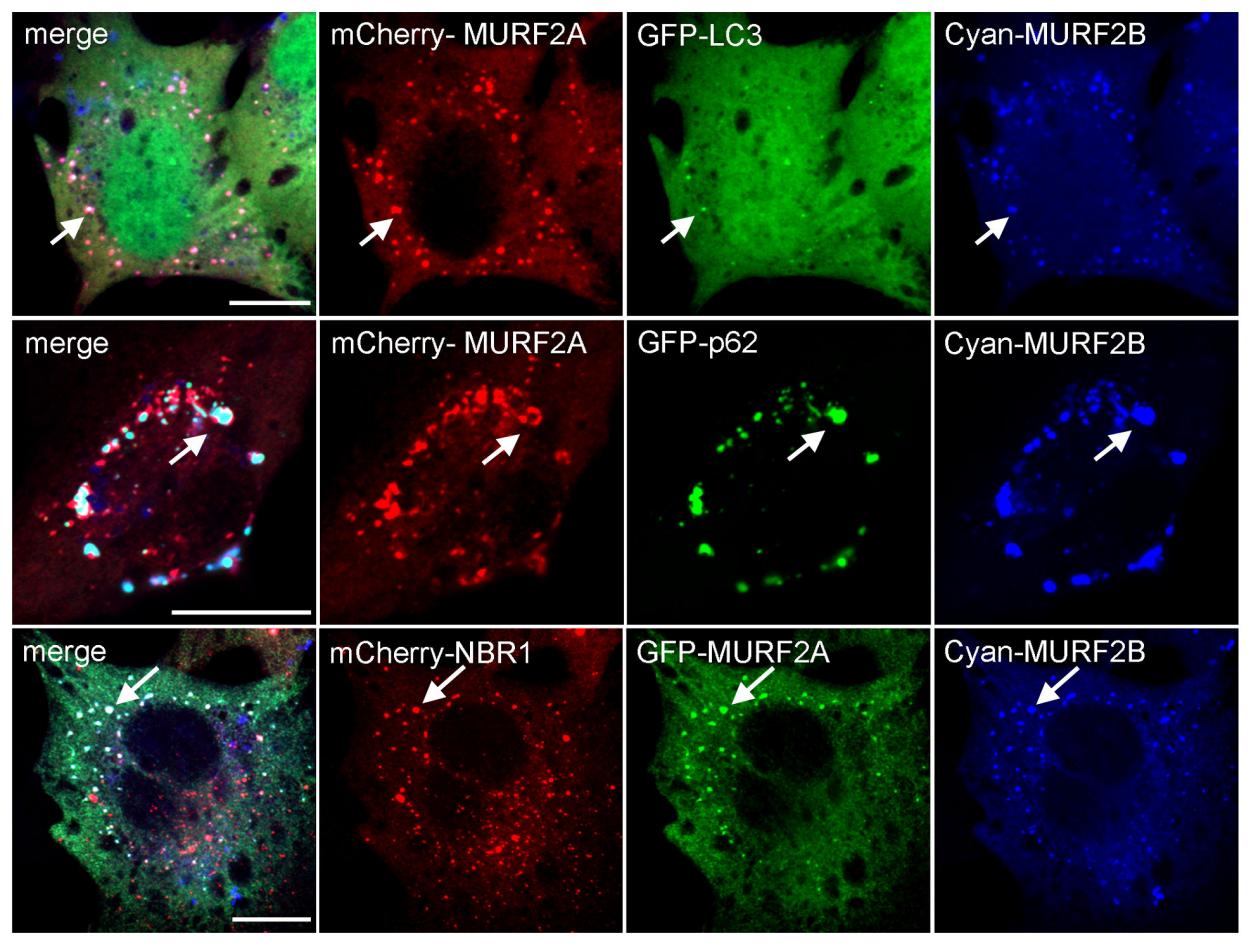
MURF2A and MURF2B colocalize with autophagic proteins. Triple co-transfections were performed in C2C12 myoblasts with combinations of various plasmids encoding the mentioned tagged proteins. Direct observation of fluorescent proteins was performed by confocal microscopy. Arrows show colocalization of the proteins on vesicles and aggregates. Scale Bar: 10 µm.

By performing two-hybrid and biochemical experiments using rat cardiac muscle extracts, it was reported that MURF2A interacts with p62 [19] and that during skeletal muscle development and C2C12 differentiation, expression of MURF2A parallels that of proteins involved in autophagy, namely LC3, p62 and NBR1 [16]. Since parallel localization of MURF2 isoforms with LC3, p62 and NBR1 had not been examined so far in C2C12 cells, we examined these locations. All available antibodies needed to identify MURF2, LC3 and p62 mouse endogenous proteins were raised in rabbits, therefore double immunofluorescence experiments were performed with fluorescent tag proteins (Figure 2). Only cells expressing low level of exogenous proteins were analyzed to avoid artefactual aggregation due to protein overexpression. When GFP-MURF2A was co-expressed with mCherry-p62 in C2C12 in myoblasts, nearly all the vesicles decorated with p62 were in close proximity with a cytoplasmic reticular structure labelled by MURF2A (Figure 2, enlarged observations). After overexpression of GFP-LC3, LC3 vesicles did not show extensive colocalization with the reticular structure labelled by mCherry-MURF2A. Expression of GFP-MURF2A and mCherry-NBR1 revealed that some vesicles were decorated with NBR1 and MURF2A. Analysis of MURF2B revealed that it colocalized perfectly with vesicles decorated with p62, NBR1 and LC3 (Figure 2, enlarged observations). Ring proteins are implicated in the formation of macromolecular assemblages and the integrity of the RING domain is needed for the formation of the complexes [21,22]. Thus, to confirm our results, we constructed MURF2 mutants impeded in their ubiquitin ligase activity by replacing cysteine 29 and 78 of the RING Zinc-finger domain by alanine, as previously described [19]. Using the fluorescent tag MURF2A mutant (designated MURF2A Ubi) for double double immunofluorescence experiments, we observed that MURF2A Ubi displayed a blurred filamentous labelling that did not colocalize with NBR1 (Figure S2). When the MURF2B mutant (MURF2B Ubi) was used, it appeared that MURF2B Ubi did not colocalize anymore with vesicles decorated with p62, LC3 and NBR1 (Figure S2). Since MURF2 proteins could form homodimers and heterodimers as observed for MURF1 and MURF3 [23] and that heterodimerization could mediate diverse protein interactions, we analyzed if co-expression of both MURF2 isoforms could modify their locations in regard to autophagic proteins. Results of triple transfections, performed in C2C12 myoblasts, show that co-expression of MURF2A and MURF2B with LC3, p62 or NBR1 resulted in positioning all the exogenous proteins on vesicles and aggregates (Figure 3). Upon inhibition of UPS, ubiquitinylated proteins can accumulate to a storage compartment, the aggresome [24]. Aggresome forms at the microtubule-organizing center and is accompanied by the disruption of the vimentin network [25]. To exclude that the structures we observed after overexpression of exogenous proteins could be aggresome bodies, we investigated the vimentin network of C2C12 cells expressing GFP-vimentin together with the mCherry- MURF2 isoforms or their Ubi mutants (Figure S3, double transfection). Compared to cell transfected only with GFP-vimentin (Figure S3, single transfection), the double transfected cells did not show significant rearrangement of the vimentin network, suggesting that the structures observed in Figure 2 and Figure S2 were not aggresome bodies. All these results show that in myoblasts, overexpressed MURF2A does not localize with vesicles decorated with p62 and LC3 but localizes to some extent with NBR1 vesicles. In contrast, overexpressed MURF2B colocalizes with vesicles decorated with p62, LC3 and NBR1. Vesicular location of MURF2A and MURF2B depends on their ubiquitin ligase activity. Furthermore joint expression of the MURF2 isoforms results in the relocation of MURF2A on vesicles or aggregates labelled with p62, LC3, NBR1 and MURF2B.

### MURF2B is a novel LC3-interacting protein

**Figure 4:**
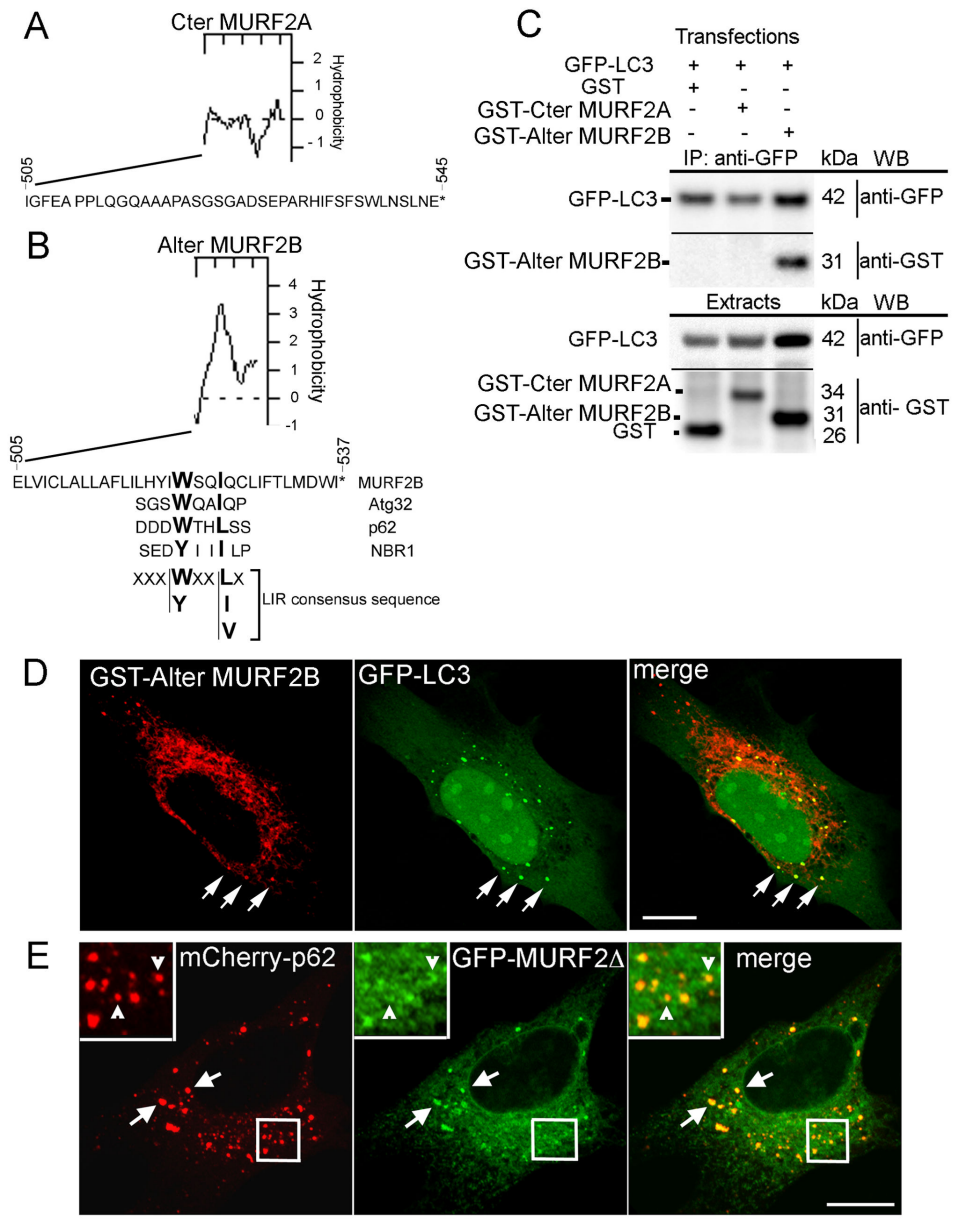
A LIR sequence mediates interaction of MURF2B with LC3. (A,B) Predicted hydrophobicity and aa sequences of the human C-terminus of MURF2A (A, Cter MURF2A) and of the Alternative C-terminus of MURF2B (B, Alter MURF2B). LIR sequences of various proteins and consensus sequence are aligned with the MURF2B C-terminus. (C) The MURF2B LIR domain interacts with LC3. C2C12 myoblasts were transfected with various pairs of vectors encoding GFP-LC3, GST, GST- MURF2A C-terminus domain (GST- Cter MURF2A) or the GST- MURF2B Alternative C-terminus domain (GST-Alter MURF2B). One day after transfection, cells were differentiated for 24h day in 1% HS medium then cultured for 2h 30 min in HBSS medium containing 0.1% BSA, 50 µM MG132 and 200 nM Baf. Immunoprecipitation were performed with GFP-Trap magnetic beads and 400 µg of extracts in RIPA buffer and subjected to SDS-PAGE (IP: anti-GFP). Exogenous proteins were detected with anti-GFP and anti-GST antibodies by Western blot (WB). For each transfection, 20 µg protein extract was used to reveal the expression of exogenous proteins (Extracts). (D) The Alternative C-terminus domain of MURF2B interacts with vesicles decorated with LC3 and the C-terminus domains of the MURF2 isoforms are not needed for MURF2/p62 interactions. Immunofluorescence experiments were performed with C2C12 cells transfected with GST-Alter MURF2B and GFP-LC3 vectors and stained with anti-GST antibody (red). GFP-LC3 was observed directly (green) with confocal microscope. Co-localizations are highlighted by arrows. (E) C2C12 cells were also transfected with the GFP-MURF2Δ and mCherry-p62 vectors and observed by confocal microscopy. Arrows show vesicles displaying both exogenous proteins. Enlarged views reveal curved structures, labelled with GFP-MURF2Δ, in close contact with p62 vesicles (empty arrowheads). Scale Bars: 10 µm.

MURF2A and MURF2B differ by their C-terminus domains (Figure 1A and Figure 4A-B). Hydrophobicity analysis suggested that these regions could behave differently, MURF2B displaying a very hydrophobic C-terminus as opposed to MURF2A. Moreover, in the C-terminus domain of MURF2B (Figure 1A, Alter MURF2B) we detected a sequence (aa 522-526) that presents homology with the consensus sequence of the LC3-interacting region (LIR) present in various proteins (Figure 4B) [26]. We established that the MURF2B LIR motif was functional by performing immunoprecipitation experiments using the GST-tagged Cter MURF2A (aa 505-545) and the GST-tagged Alter MURF2B (505-537) domains (Figure 4C). The GST protein was used as control in this experiment. This result was confirmed by immunofluorescence experiments using C2C12 cells transfected with vectors encoding GFP-LC3 and the GST-Alter MURF2B domain that contains the LIR motif (Figure 4D). To determine if the MURF2B/p62 association depends on the MURF2B LIR motif, immunofluorescence experiments were performed by co-expressing a GFP-MURF2 protein deleted of its C-terminus domain (MURF2∆) with mCherry-p62 (Figure 4E). In addition to numerous vesicles labelled with the two exogenous proteins (Figure 4E, arrows), some mCherry-p62 vesicles co-localized partially with crescent-shaped structures decorated with MURF2∆ (Figure 4E, arrowheads in enlarged view).

Our results show that MURF2B can mediate LC3 interaction via a LIR sequence located at the C-terminus of the protein and that this sequence is not essential for MURF2B/p62 interaction. Thus, p62 interaction with MURF2A or MURF2B is mediated by a protein domain common to both isoforms.

### During myogenic differentiation, MURF2 proteins can form two types of complexes with p62 and LC3.

**Figure 5:**
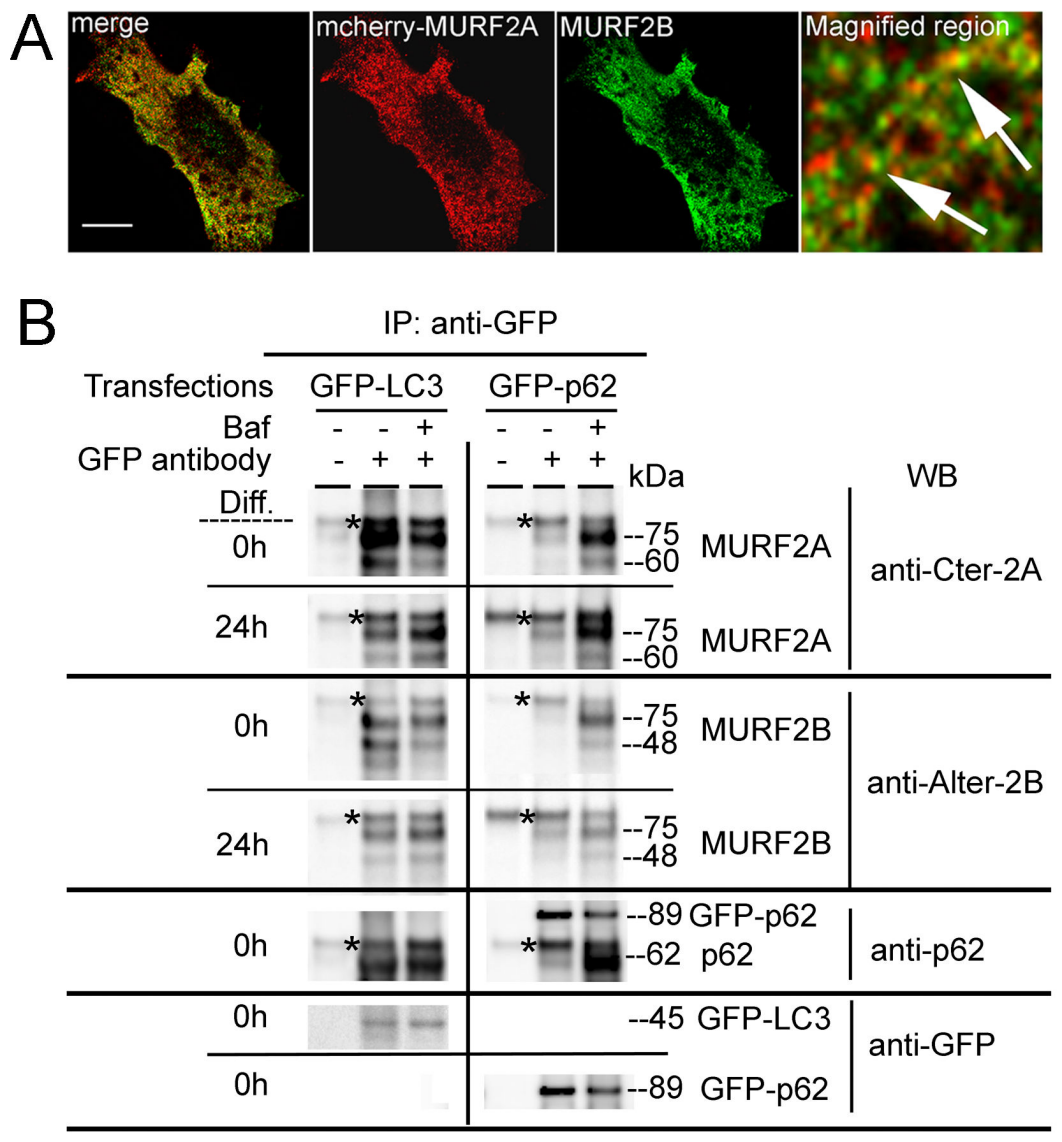
During myogenic differentiation, various MURF2 complexes are formed. (A) MURF2A and MURF2B co-localize in C2C12 myoblasts. C2C12 cells were transfected with mCherry-MURF2A. After 24h, endogenous MURF2B was detected using MURF2B antibody (green) and fluorescent MURF2A was observed directly (red). The magnified region reveals that MURF2A and MURF2B are in close contact (arrows). Scale Bars: 10 µm. (B) MURF2A and MURF2B are complexed with LC3 and p62. C2C12 cells transfected with GFP-LC3 or GFP-p62 were kept undifferentiated (Diff. 0h) or differentiated for 24h (Diff. 24h) in absence (-) or in presence (+) of 200 nM Baf for 4 h before cell lysis in RIPA buffer. Western blots were performed with precipitated proteins obtained using magnetic beads (Bio-Adembeads pAG) incubated with 300 µg extract complemented (+) or not (-) with anti-GFP antibody. Western blots (WB) were probed with the indicated antibodies. Asterisks indicate unspecific signals.

Given that MURF2A colocalization with p62 and LC3 on vesicular structures depends on the overexpression of MURF2B (Figure 3), that MURF2B can bind LC3 with its LIR domain (Figure 4) and that expression of both MURF2 isoforms is specifically regulated during myogenic differentiation (Figure 1), we examined whether endogenous MURF2A and MURF2B could form specific complexes with p62 and LC3 according to the differentiated state of the C2C12 cells. First we examined the location of endogenous MURF2B and of low expressed mCherry-MURF2A in myoblasts. After transfection of C2C12 cells with mCherry-MURF2A, this protein was observed directly (red) and MURF2B was detected with the Alter-2B antibody (green) (Figure 5A). The magnified region showed that MURF2A and MURF2B were in close contact not on vesicles but on reticular structures (Figure 5A, arrows). Albeit the lack of vesicular structures decorated with MURF2 isoforms, these proteins could be engaged in autophagy. Indeed, during the first step of autophagy, p62 targets to the phagophore formation sites independently of LC3, then p62 incorporates into autophagosomes in an LC3-dependent manner [27]. Later p62 is degraded, with the substrates it delivers, into autophagosomes when they fuse with lysosomes to form autolysosomes [9]. The autophagosome-lysosome fusion and the lysosomal degradation can be inhibited by Bafilomycin A1 (Baf) [28]. Thus, we examined whether endogenous MURF2A and MURF2B could form heterodimers in complexes with GFP-p62 or GFP-LC3 expressed in C2C12 myoblasts and in myotubes (Figure 5B). In order to discriminate the protein complexes engaged in late autophagic stages, the transfected myoblasts and myotubes were treated or not with Baf for 4 h before cell lysis and immunoprecipitation experiments performed with magnetic beads coated with rabbit anti-IgG antibodies after addition of anti-GFP antibody to cell lysats (Figure 5B, GFP antibody +). As a negative control, no GFP antibody was added in the cell lysats (Figure 5B, GFP antibody -). In myoblasts expressing GFP-LC3 (Figure 5B, Diff. 0h), LC3 formed complexes with MURF2A, MURF2B and p62 in the absence of Baf. Since very low amounts of MURF2A are present in myoblasts (Figure 1B), nearly all MURF2A protein must be in complexes. Surprisingly, when Baf was added, the amounts of MURF2A and MURF2B associated with GFP-LC3 decreased, whereas endogenous p62 increased as expected after inhibition of its autophagic degradation. Since LC3 remains associated with autophagic membranes all along autophagy [8], the MURF2 proteins associated with LC3 could form Baf-insensitive complexes engaged in the early steps of autophagy, namely phagophore formation or other types of complexes not degraded by autophagy. In myotubes (Figure 5B, Diff. 24h), we observed that both MURF2 proteins formed complexes with GFP-LC3 and that the amount of the proteins in the complexes increased after Baf treatment. In myoblasts and in myotubes expressing GFP-p62 in the absence of Baf, MURF2A and MURF2B were barely detectable in the immunoprecipitated complexes although GFP-p62 immunoprecipitation was efficient. When myoblasts and myotubes were treated with Baf, the level of MURF2 and of endogenous p62 complexed with GFP-p62 increased considerably.

Collectively, our results indicate that in C2C12 myoblasts most MURF2 and LC3 proteins can form Baf-insensitive complexes suggesting that these complexes could be resident of pre-autophagosome structures or in structures not involved in autophagy. In myoblasts, the MURF2/p62 Baf-sensitive complexes should refect a basal autophagic activity of the growing cells. In myotubes, MURF2A and MURF2B establish Baf-sensitive complexes with p62 and LC3 suggesting that the complexes must be residents of autophagosmes and autolysosomes.

### Analysis of autophagy during differentiation of C2C12 cells

**Figure 6:**
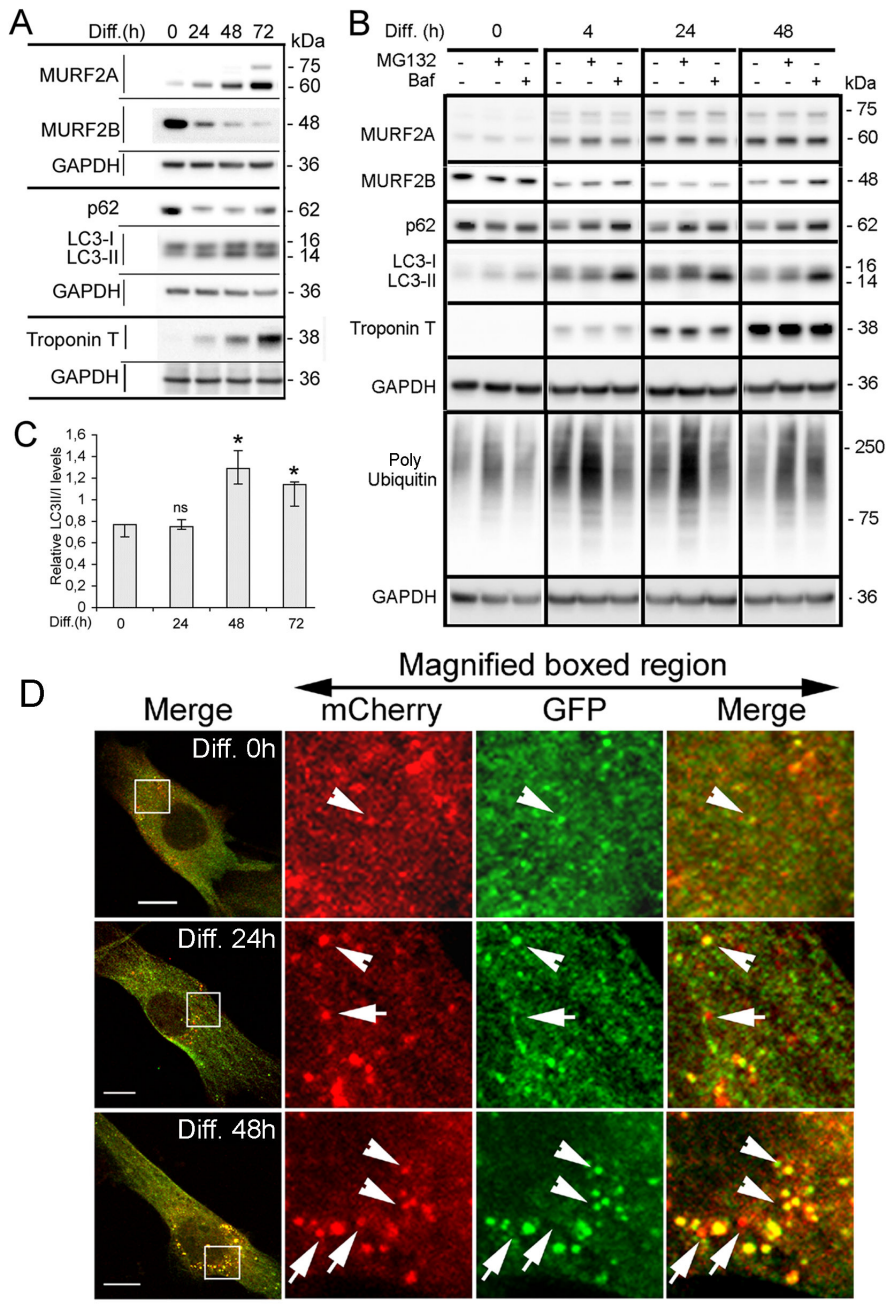
MURF2A and MURF2B are needed for autophagosome formation. (A) Expression of MURF2, p62 and LC3 during C2C12 differentiation. Representative immunoblots of C2C12 lysates probed with MURF2, p62, LC3, Troponin T and GAPDH antibodies. Lysates were obtained at various times of differentiation (Diff.). (B) Analysis of autophagy and polyubiquitination during differentiation. Representative immunoblots of C2C12 lysates obtained at various times of differentiation (Diff.) in absence (-) or in presence (+) of inhibitors (50 µM MG132 or 200 nM Baf) applied during 4h. Western blots were probed with the antibodies mentioned. (C) Quantification of the ratio of LC3-II/LC3-I proteins during differentiation. LC3-II and LC3-I detected with 3 independent Western blot analyses were quantified. Differences in LC3-II/LC3-I ratio were evaluated with the Kruskal-Wallis test (analysis of variance). Histograms show medians and quartiles of LC3-II/LC3-I expression during differentiation of C2C12 cells. Compared to undifferentiated cells (Diff. 0), non significant (ns) and significant (*) differences were observed (P<0.05). (D) Autophagic flux increases after 48h of differentiation. Stable C2C12 cells expressing mCherry-GFP-LC3 were induced to differentiate for the indicated times and fluorescent LC3 vesicles observed directly by confocal microscopy. Magnified regions show yellow autophagosomes (arrowheads) and red autolysosomes (arrows). Scale Bar: 10 µm.

Although it has been shown that MURF2A isoforms expression parallels that of LC3 and p62 proteins during C2C12 cells differentiation [16], no relation with autophagic activity had been reported. To reveal activation of autophagy, variations of p62 and LC3 proteins can be studied. Indeed, p62 is degraded inside autolysosomes [9] and therefore diminished upon normal autophagic activation. Likewise, after its synthesis, LC3 is cleaved to form LC3-I that conjugates with PE to yield LC3-II that associates with autophagosomes [29]. Thus, increased levels of LC3-II compared to LC3-I can reveal activation of autophagy. By Western blot analysis we examined the expression of MURF2 isoforms, p62, LC3-I and LC3-II, as differentiation of C2C12 progressed (Figure 6A). As previously observed (Figure 1A) MURF2A and MURF2B displayed antagonistic expression and comparable amounts of both isoforms were detected around 24h of differentiation. In myoblasts (Figure 6A, Diff. 0h), the amount of LC3-II was very low compared to LC3-I and the amount of p62 was higher than in differentiated cells. These observations suggested a weak basal autophagic activity in myoblasts. After differentiation was initiated by serum withdrawal, LC3-II expression increased and the amount of p62 decreased during the first 48h, suggesting that autophagy could be stimulated. After 72h of differentiation, in myotubes displaying high levels of Troponin T, expression of LC3-II decreased and p62 increased indicating that the autophagic flux could be slowed down. The accumulation of LC3-II and the decrease of p62 observed during the first 48h of differentiation could reflect an increased production of autophagosomes, a block in the autophagic flux or the degradation of proteins involved in autophagic complexes by UPS. To discriminate between these possibilities, we examined the effects of MG132 and Baf that respectively inhibit UPS and autophagy on the expression of MURF2, LC3 and p62 during C2C12 differentiation (Figure 6B). For each time point of differentiation expression of the proteins in the absence or in the presence of each inhibitor was compared. In myoblasts (Figure 6B, Diff. 0h), neither MG132 nor Baf induced significant increase of the proteins, although MG132 was effective as revealed by an increase of polyubiquitinylated proteins. As early as 4h after differentiation started, the amount of p62 increased when MG132 was applied, but no changes of LC3 and MURF2 could be revealed. Identical results were obtained when MG132 was used at later stages of differentiation. When Baf was applied at all the time points of differentiation, a high increase in LC3-II and p62 was detected. MURF2A did not seem to be affected by addition of Baf, whereas MURF2B proteins increased after 48h of differentiation. These results indicated that myoblasts display low autophagic activities and that autophagy was stimulated upon differentiation. To confirm our results, the ratio of LC3-II versus LC3-I was evaluated at different times of differentiation. Quantifications of LC3-II/I levels were obtained from three independent Western blot experiments, as presented in Figure 6A. The relative levels of LC3-II/I showed that autophagy reached a maximum after 48h of differentiation and diminished thereafter (Figure 6C). To follow the autophagic flux during differentiation, we also performed confocal microscopy observations of stable C2C12 cells expressing the double tag mCherry-GFP-LC3 protein. This reporter protein [10] displays green and red fluorescence (yellow as merged image) when linked to nonacidic phagophores and autophagosomes, but only red labelling when associated to autolysosomes due to the quenching of GFP in these acidic structures. We observed that myoblasts expressing mCherry-GFP-LC3 displayed very few small yellow vesicles (Figure 6D, Diff. 0h, arrowheads). After 24h of differentiation, autophagosomes (Figure 6D, Diff. 24h, arrowheads) and autolysosomes (Figure 6D, Diff. 24h, arrows) started to form. After 48 h of differentiation (Figure 6D, Diff. 48h) the number of autophagosomes (arrowheads) and autolysosomes (arrows) increased indicating that autophagy was activated.

Taken together, these results show that autophagy is strongly activated in C2C12, after two days of differentiation when both MURF2A and MURF2B are expressed in the same range.

### MURF2A and MURF2B are needed for autophagosome formation

**Figure 7:**
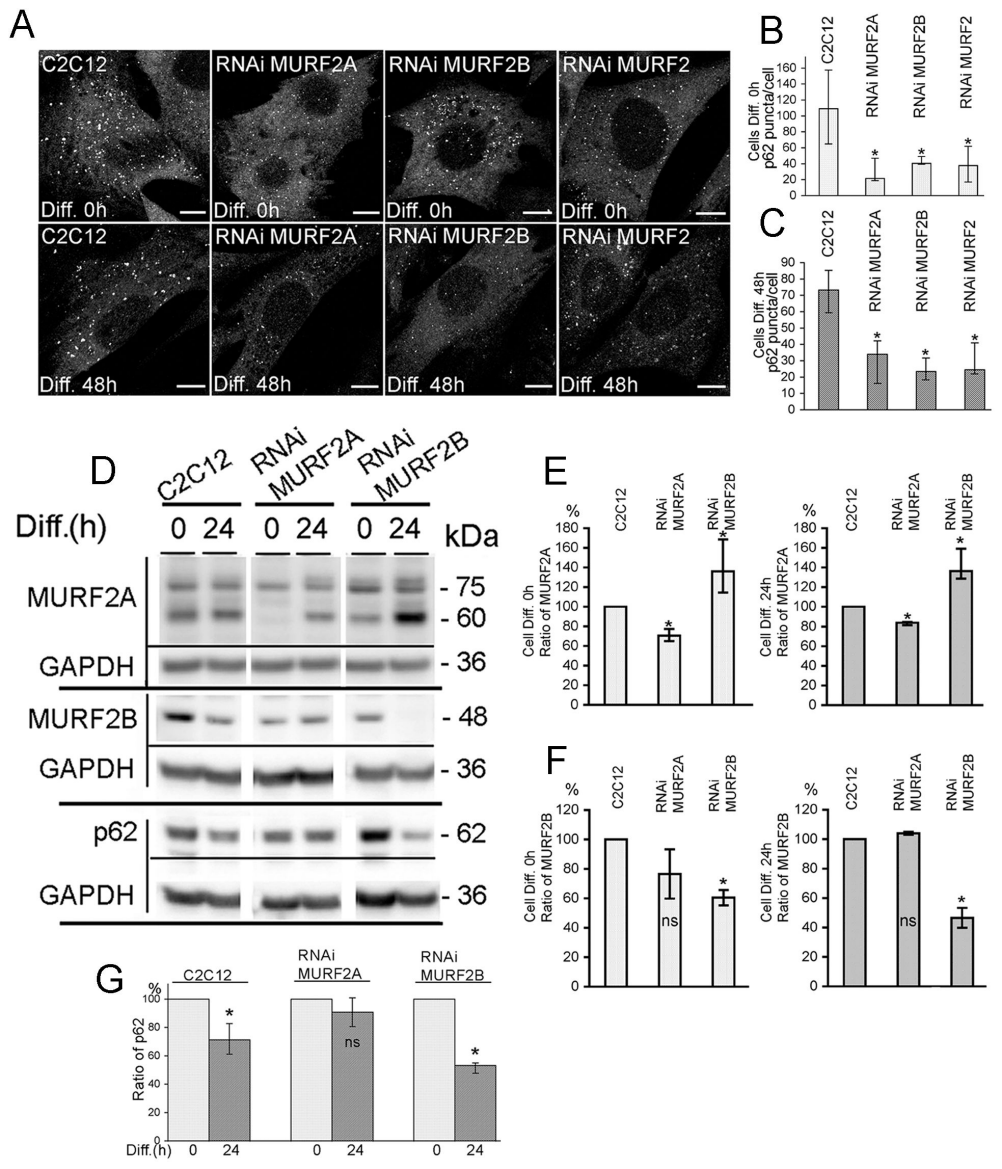
Analysis of autophagy in C2C12 and MURF2 RNAi cells. (A) Endogenous p62 was detected in C2C12 cells and MURF2 RNAi myoblasts (Diff. 0h) and in cells differentiated for 48h. Cells were immunostained with anti-p62 antibody and analyzed by confocal microscopy. Scale Bar: 10 µm. (B) Quantification of the p62 puncta detected in C2C12 and MURF2 RNAi myoblasts. Data were obtained from independent imunofluorescence experiments and various numbers of cells were analyzed : 8 for C2C12 cells, 6 for RNAi MURF2A cells, 5 for RNAi MURF2B cells and 13 for RNAi MURF2 cells. Differences were evaluated by the Kruskal-Wallis test. Medians and quartiles are shown in the histograms. Compared to C2C12 myoblasts, the MURF2 RNAi cells showed significant differences (*, P<0.05). (C) Quantification of the p62 puncta in the C2C12 and MURF2 RNAi 2 after 48h of differentiation. Data were obtained from independent imunofluorescence experiments, and various numbers of cells were analyzed: 12 for C2C12 cells, 24 for RNAi MURF2A cells, 9 for RNAi MURF2B cells and 12 for RNAi MURF2 cells. Medians and quartiles ranges are shown in the histograms. Multiple comparaisons of median of C2C12 and MURF2 RNAi cells by the Kruskal-Wallis test showed significant differences (*, P<0.05). (D) Representative immunoblot analyses of endogenous proteins expressed during differentiation of C2C12 and MURF2 RNAi cells. Lysates were obtained from myoblasts (Diff. 0) and after 24h of differentiation. Western blots were probed with the indicated antibodies. (E) Quantification of MURF2A protein in C2C12 and MURF2 RNAi cells after 0h and 24h of differentiation. For each time point of differentiation, data were obtained from 3 independent western blot experiments. Medians and quartiles ranges are shown in the histograms. Multiple comparaisons of median of C2C12 and MURF2 RNAi cells by the Kruskal-Wallis test showed significant differences (*, P<0.05). (F) Quantification of MURF2B protein in the C2C12 and MURF2 RNAi cells after 0h and 24h of differentiation. For each time point of differentiation, data were obtained from 2 independent western blot experiments. Medians and quartiles ranges are shown in the histograms. Multiple comparaisons of median of C2C12 and MURF2 RNAi cells by the Kruskal-Wallis test showed significant differences (*, P<0.05). (G) Endogenous p62 quantification in undifferentiated (Diff. 0h) and differentiated (Diff. 24h) C2C12 and MURF2 RNAi cells. Cells were lysed with RIPA buffer and analyzed by Western blots with anti-p62 antibody. For each cell line, the amounts of p62 detected after differentiation were normalized with p62 present in undifferentiated cells. Data were obtained from several independent Western blot experiments: 6 for C2C12 cells (Diff. 0h and 24h), 3 for RNAi MURF2A (Diff. 0h), 2 for RNAi MURF2A (Diff. 24h) and 3 for RNAi MURF2B (Diff. 0h and 24h). Differences were evaluated with the Kruskal-Wallis test. Medians and quartiles are shown in the histograms. Compared to undifferentiated cells (Diff. 0h), non significant differences (ns) and significant differences (*) were observed (P<0.05).

**Figure 8:**
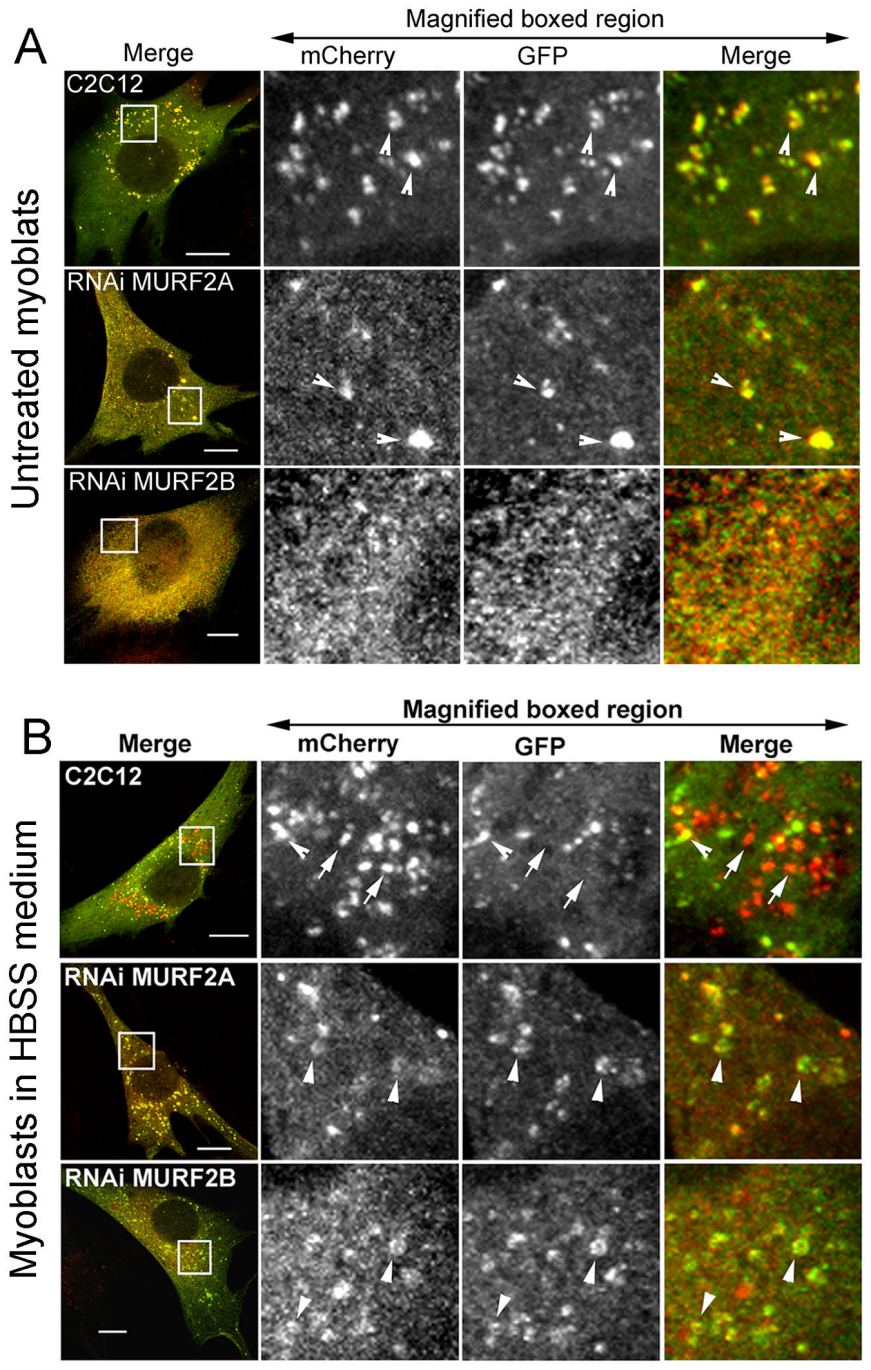
MURF2A activates the 20S proteasome and is implicated in p62 degradation. (A) Undifferentiated stable C2C12 and RNAi cell lines expressing the mCherry-GFP-LC3 protein were grown in 10% FCS and observed directly by confocal microscopy. In magnified regions, the yellow autophagosomes are indicated by empty arrowheads. (B) The same cell lines were kept in HBSS medium for 45 min and observed directly by confocal microscopy. Magnified regions show in C2C12 cells yellow autophagosomes (empty arrowheads) and red autolysosomes (arrows). In RNAi cells unconventional autophagic structures are indicated by arrowheads. Scale Bar: 10 µm.

To shed some light on the specific roles of MURF2 isoforms in the autophagic machinery, MURF2 proteins were repressed by RNA interference through the expression of short hairpin RNAs (shRNA). MURF2 RNAi subclones of C2C12 cells expressing doxycycline-inducible shRNAs specific for mouse MURF2A (RNAi MURF2A cells), MURF2B (RNAi MURF2B cells) or for all MURF2 proteins (RNAi MURF2 cells) were established. We verified by Western blots experiments that expression of the endogenous MURF2 proteins could be modulated during differentiation upon addition of doxycycline (Dox) to the cell medium (Figure S4A). MURF2 proteins were greatly reduced in the RNAi cell lines after 72 hours of differentiation in the presence of Dox. However, we also observed that in RNAi MURF2 cells, expression of the MURF2 isoforms was reduced even without Dox, indicating that the promoter of the shRNA vectors must not be fully repressed in the absence of Dox. Since inhibition of MURF2 expression was maximum after three days of Dox treatment, a period of time needed to accumulate enough shRNA within the cells, we always cultivated cells with Dox for three days before performing experiments when MURF2 expression had to be inhibited (e.g. Figure 7D). We confirmed that shRNAs were effective and specific by immunofluorescence experiments performed on C2C12 and RNAi cells. Since MURF2B is expressed mostly in myoblasts (Diff. 0h) and MURF2A in myotubes, immunofluorescence experiments were performed on C2C12 and RNAi myoblasts with the Alter-2B antibody (Figure S4B) and after 48h of differentiation with the Cter-2A antibody (Figure S4C). When endogenous p62 was revealed by immunofluorescence experiments, C2C12 myoblasts presented more p62 vesicles than the various undifferentiated MURF2 RNAi cells (Figure 7A, Diff. 0h). This was confirmed by quantifying the vesicles (Figure 7B). After 48h of differentiation, all RNAi cells displayed fewer p62 puncta than C2C12 myotubes (Figure 7A, Diff. 48h). Quantification of the p62 puncta confirmed imunofluorescence observations (Figure 7C). Expression of MURF2 and p62 was determined in these cells. Figure 7D is representative of several Western blot analyses. Quantification of the Western blots revealed that, as expected, the amount of MURF2A was significantly reduced by 30% in undifferentiated and differentiated RNAi MURF2A. In contrast, in the RNAi MURF2B myoblasts (Diff. 0h) and myotubes (Diff. 24h), the level of MURF2A was about 40% higher than the one detected in C2C12 cells (Figure 7E). Quantification of MURF2B (Figure 7F) showed as expected that the amount of MURF2B was significantly decreased in RNAi MURF2B cells, around 40% in myoblasts and 50% after 24h of differentiation. For p62, expression of the protein detected by Western blots was quantified for each cell line, and the ratio of p62 detected between myoblasts and myotubes was established (Figure 7G). Upon differentiation, p62 decreased by 25% in C2C12 cells and by 48% in RNAi MURF2B cells. Surprisingly, the amount of p62 expressed in the RNAi MURF2A cells remained high after differentiation whereas only few p62 vesicles were detected in these myotubes (Figure 7A Diff. 48h and 7C). Since p62 with LC3 is needed for nucleation and elongation of the phagophore [8,27], we investigated whether the decrease of p62 vesicles observed in RNAi cells could result from activation of autophagy leading to p62 degradation, or whether it could reflect impairment of autophagosome formation. Therefore, the autophagic flux was examined in stable C2C12 and RNAi cell lines expressing the mCherry-GFP-LC3 construct. In myoblasts few yellow autophagosomes were detected in C2C12 cells and almost none in RNAi cells (Figure 8A, empty arrowheads). When myoblasts were kept in HBSS medium for 45 min to stimulate autophagy, the C2C12 cells displayed yellow autophagosomes (Figure 8B, empty arrowheads) and numerous red autolysosomes (Figure 8B, arrows). In contrast, MURF2 RNAi cells displayed very few red vesicles and most of the yellow labellings observed appeared as yellow-rimed hollow structures (Figure 8B, arrowheads). Altogether, these results indicate that in the absence of MURF2A and in the presence of MURF2B, expression of p62 is unchanged in myoblasts and in myotubes but autophagic structures do not form normally. In the absence of MURF2B and in the presence of MURF2A, the levels of p62 diminish extensively in myotubes and autophagic vesicle formation is impaired. Therefore both MURF2 isoforms are needed for normal autophagy in C2C12 cells.

### MURF2A is implicated in UPS activation and degradation of p62

**Figure 9:**
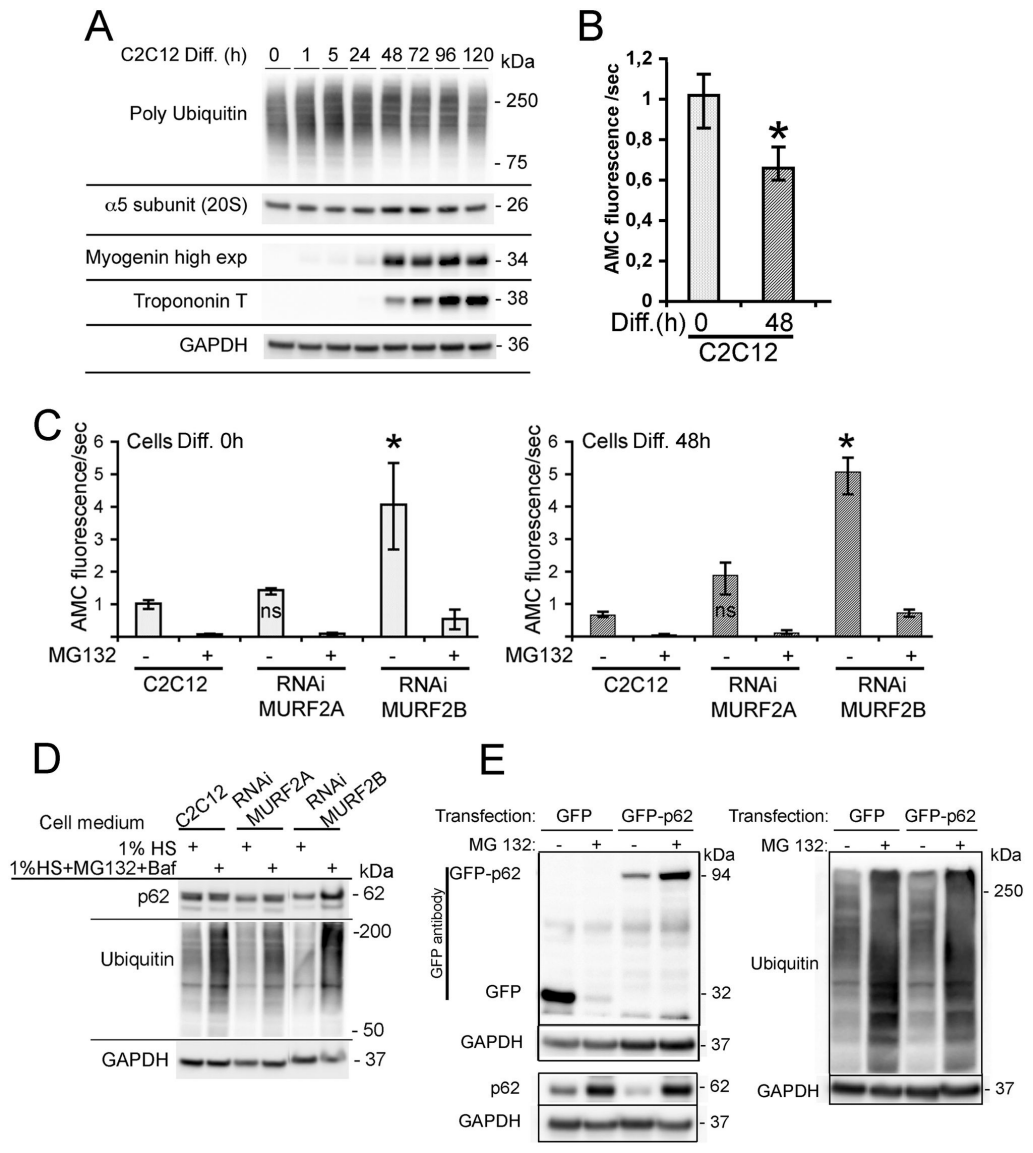
(A) Representative immunoblots of C2C12 lysates probed with poly ubiquitin, the α5 subunit of the 20S proteasome, myogenin, Troponin T and GAPDH antibodies. Lysates were obtained at various times of differentiation (Diff.). (B) Activity of the 20S proteasome in C2C12 cells. Proteolytic 20S proteasome chymotrypsin-like activity of the indicated undifferentiated (0h) and differentiated (48h) C2C12 cells was determined measuring the amidomethylcoumarin (AMC) generated as the cleavage product of the fluorogenic substrat succinyl-leucine-leucine-valine-tyrosine-7 amido-4-methyl-coumarin. Differences were evaluated by the Kruskal-Wallis test. Medians and quartiles are shown in the histograms. Compared to C2C12 myoblasts (Diff. 0h), myotubes (Diff. 48h) showed significant differences (*, P<0.05). (C) Activity of the 20S proteasome in various RNAi cell lines. The proteolytic 20S proteasome chymotrypsin-like activity was determined for undifferentiated (0h) and differentiated (48h) cells as previously mentioned for C2C12 cells. Compared to C2C12 and RNAi MURF2A, RNAi MURF2B cells showed significant increase of their 20S proteasome activity (*, P<0.05). (D) Effect of UPS and autophagic inhibition on p62 expression in C2C12 and MURF2 RNAi cells. After 24h of differentiation in 1% horse serum medium (1% HS), cells were shifted to fresh differentiation medium (1% HS) or treated for 2h 30min with fresh differentiation medium complemented with 50 µM MG132 and 200 nM Baf (1% HS+MG132+Baf) before lysis. Western blots were probed with anti p62, anti mono and poly ubiquitin and anti GAPDH antibodies. (E) p62 is degraded by UPS in differentiated C2C12 cells. C2C12 cells were transfected with vectors encoding GFP or GFP-p62 under the CMV promoter. 24h after transfection, cells were split into two groups, induced to differentiate for 24h, then shifted to fresh differentiation medium containing (+) or not (-) 10 µM MG132 for 12h. Extracts were used for Western blot analyses and probed with the indicated antibodies. Rabbit anti mono and poly ubiquitin antibody was used.

**Figure 10:**
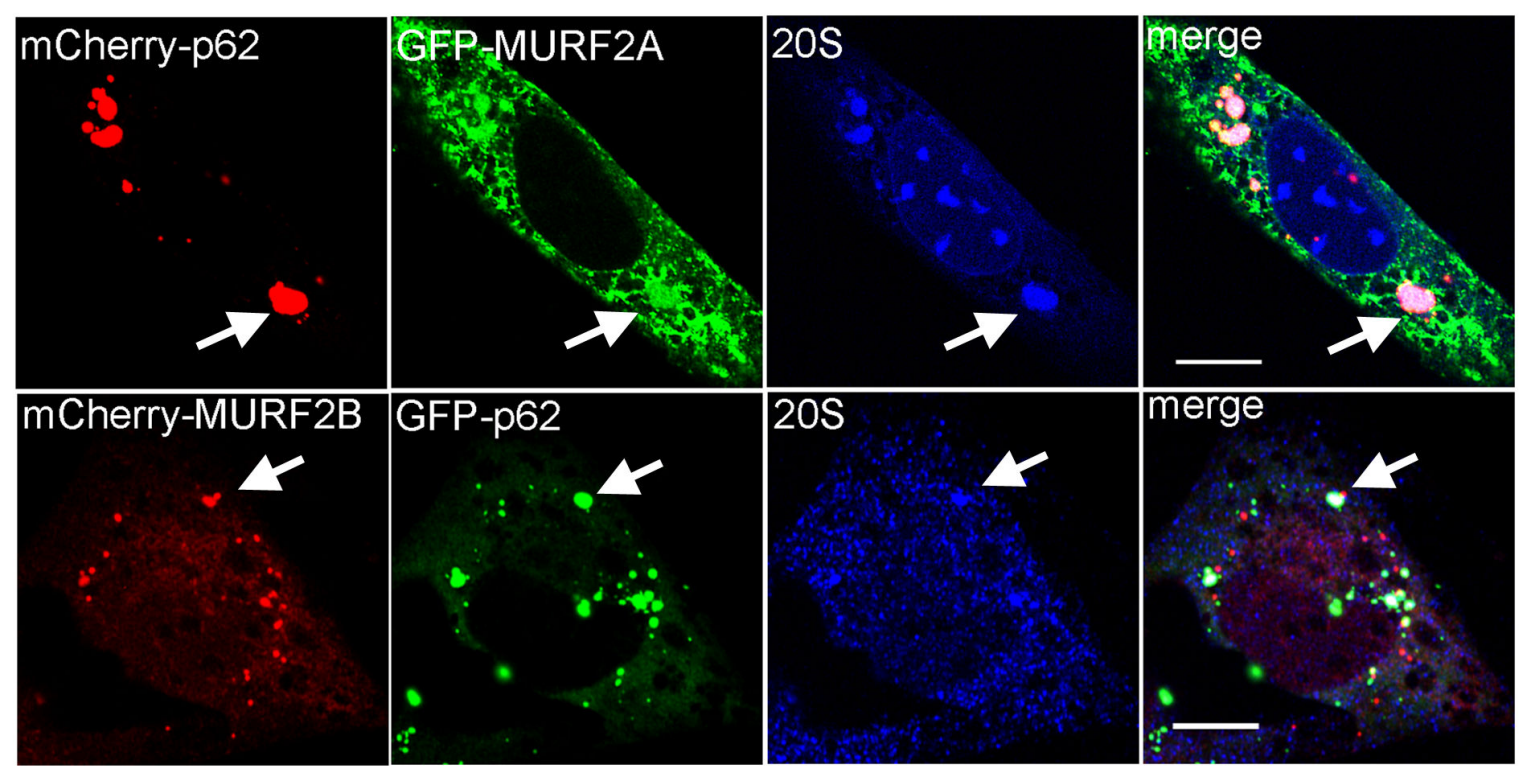
Confocal analyses of p62, UPS and MURF2 proteins in differentiated C2C12 cells. C2C12 cells were transfected with plasmids expressing the fluorescent proteins as indicated and differentiated for 24h. Confocal observations: fluorescent proteins were detected directly and the 20S proteasome subunit revealed by specific antibody for the α5 subunit of the 20S proteasome. Scale Bars: 10 µm.

Our previous results suggested that p62 could be degraded by a MURF2A-dependent UPS pathway. Indeed, in RNAi MURF2B myotubes, which only express MURF2A and are devoided of autophagic flux, low levels of p62 were detected (Figures 7G, 24h). In C2C12 myoblasts that only express very low level of MURF2A (Figures 6A, 0h), a high level of p62 was revealed (Figures 7D, 0h). In myotubes, the amount of p62 increases after addition of MG132 (Figure 6B). Therefore, we investigated whether MURF2A could participate in the UPS degradation of p62. When the pattern of polyubiquitinated proteins was analyzed during differentiation of C2C12 cells by using the same cell extracts of Figure 1B, we detected an increase of ubiquitination during the first hours of differentiation and a decrease in fully differentiated myotubes (Figures 9A, 120h). Since accumulation of polyUb proteins could reflect either an increase or an inhibition of the UPS activity and since the amount of proteasome is regulated at the transcription level and subject to an autoregulatory feedback mechanism [30], the increase of polyubiquitinated proteins could also reflect a decrease of the amount of proteasome. To test this hypothesis, the α5 subunit of the 20S proteasome was revealed during C2C12 differentiation (Figure 9A). No change of the α5 subunit was detected, indicating that the increase of polyubiquitinylated proteins did not result from changes in the amount of the proteasome. Increasing amount of polyubiquitinated proteins could then reflect impairment of UPS activity, as observed with addition of MG132 or stimulation of cell catabolism [31]. Therefore, we examined the activity of the 20S proteasome in undifferentiated (0h) and differentiated (48h) C2C12 cells by using the fluorogenic substratesuccinyl-leucine-leucine-valine-tyrosine-7amido-4-methyl-coumarin. The proteolytic chymotrypsin-like activity was determined by measuring the amidomethylcoumarin (AMC) generated as the cleavage product of the substrate. By comparing the 20S proteasomal activity in C2C12 myoblasts and myotubes, we established that the chymotrypsin-like activity was significanlty reduced by 30% (Figure 9B). Therefore, the increase of polyubiquitination could reflect lower UPS activity. In order to highlight the role of MURF2A in UPS degradations, we compared the activity of the 20S proteasome in C2C12 and MURF2 RNAi cells. Whereas C2C12 and RNAi MURF2A myoblasts displayed low chymotrypsin-like activity (Figure 9C, Diff. 0h), significant increased activity was detected in RNAi MURF2B myoblasts. This activity was nearly abolished upon addition of MG132, indicating that the RNAi MURF2B cells display high intrinsic 20S activity. The same results were observed when the 20S activity was measured in myotubes (Figure 9C, Diff. 48h). Since RNAi MURF2B cells compensate for the loss of MURF2B with an increase of MURF2A protein (Figure 7E and 7F), our results suggested that increased MURF2A expression could parallel the stimulation of UPS activity. Since the amount of the p62 protein was also reduced in RNAi MURF2B cells myotubes (Figure 7D-G) we analysed the participation of UPS in p62 degradation. C2C12 and in MURF2 RNAi cells were kept in differentiating medium (1% horse serum, HS) for 24h without (Figure 9D, 1% HS) or with MG132 and Baf inhibitors (Figure 9D, 1% HS+MG132+Baf). Baf was added to prevent autophagic degradation that could result from UPS inhibition. Western blot analysis showed that MG132 treatment was efficient, since an increase in ubiquitinated proteins was detected when the inhibitor was added (Figure 9D). When p62 was observed in C2C12 and RNAi MURF2A cells, the amount of p62 did not vary extensively although inhibitors were added. In contrast, after addition of inhibitors, p62 increased in RNAi MURF2B cells, suggesting that MURF2A expression can affect the degradation of p62 by UPS. To rule out the possibility that accumulation of p62 could result from transcriptional upregulation induced by MG132 [32], exogenous GFP-p62 protein expressed under the cytomegalovirus (CMV) promoter was analyzed. Figure 9E presents a Western blot performed with extracts of C2C12 cells transfected with GFP or GFP-p62 vector and differentiated for 48h in the absence or in the presence of MG132. Upon addition of MG132, ubiquitinated proteins increased as expected after UPS inhibition. Using GFP antibody, the GFP control protein was reduced whereas GFP-p62 was highly increased (Figure 9E, MG132 +). This indicates that the exogenous promoter was not stimulated by MG132 in C2C12 cells and that GFP-p62 was sensitive to UPS degradation. When the endogenous p62 was revealed in untreated myotubes (Figure 9E, MG132 -), the amount of p62 was reduced in cells expressing GFP-p62 compared to the amount of p62 expressed in GFP transfected cells. Since p62 is degraded by autophagy [9], the overall increase of cellular p62 due to GFP-p62 expression could stimulate autophagy leading to increased degradation of the endogenous p62. However, upon UPS inhibition (Figure 9E, MG132 +), endogenous p62 increased in the various transfected cells. To confirm that MURF2A could participate to p62 degradation by UPS, C2C12 cells were double transfected with fluorescent MURF2A or MURF2B and p62 vectors and induced to differentiate for 24h. The UPS compartment was identified by looking at the α5-subunit of the 20S proteasome (Figure 10). In myotubes, p62, α5-subunit and MURF2A proteins co-localized and formed large complexes (Figure 10, arrow). In contrast, when MURF2B was expressed, only few p62/MURF2B complexes were associated with the α5-subunit (Figure 10, arrow). Thus, within myotubes MURF2A localizes with p62 and the proteasome.

Collectively, these results established that the 20S proteasome activity is stimulated when cells expressed high level of MURF2A in the absence of MURF2B and that p62 degradation displayed by the RNAi MURF2B cells could be mediated by UPS.

## Discussion

Various data suggested that MURF2A could participate in different degradative pathways, namely UPS and autophagy. MURF2 proteins display the structural features of E3 ubiquitin ligases [17,18] and MURF2A was shown to target sarcomeric myofibrillar proteins for UPS degradation [33,34]. In addition, MURF2A isoforms can form complexes with the autophagic proteins p62 and NBR1 in myogenic cells [19,35]. Also during mouse embryonic skeletal muscle development, expression of MURF2A isoforms parallels the expression of p62, NBR1 and LC3 proteins [16] suggesting that they could be involved in autophagy. However, the UPS and autophagic activity of MURF2A have not been demonstrated and the closely related isoform MURF2B never analyzed. Therefore, we studied the role of each MURF2 isoform in the degradation of ubiquitinated proteins during differentiation of C2C12 cells. Analyses of MURF2 proteins showed that expression of MURF2B is developmentally controlled, as previously reported for MURF2A isoforms [16]. However, MURF2B and MURF2A display antagonistic expression since MURF2B is mainly expressed in myoblasts and MURF2A in myotubes. Basal expression of MURF2A was also detected in C2C12 myoblasts as previously reported in chick skeletal myocytes [18]. MURF2B contains a functional LIR domain that allows interaction with LC3. As expected since LC3, p62 and NBR1 can participate in the formation of the same protein complex [10,11], overexpressed MURF2B was found on vesicles decorated with p62, LC3 and NBR1. In contrast, overexpressed MURF2A co-localized only partially with vesicles containing NBR1. However, after co-expression, both MURF2 isoforms decorated autophagic vesicles. This suggests that the ratio of both proteins could play a crucial role for autophagic vesicle formation and autophagic activity. By using various MURF2 RNAi cell lines, we confirmed that both MURF2 isoforms are needed to achieve the formation of autophagosome and that in RNAi cells the autophagic flux was hampered. In addition, at the beginning of differentiation when the amounts of endogenous MURF2B and MURF2A are similar, we determined that autophagy was activated since the LC3-II protein increased. Autophagy is a dynamic process displaying three stages: first the formation of phagophores, followed by the formation of autophagosomes and eventually the formation of autolysosomes [5]. Recent observations suggest that during phagophore formation, p62 and NBR1 localize at the ER sites independently of LC3, and that p62 triggers incorporation of LC3 into autophagosomes [27]. By analysing the complexes formed in myoblasts and in myotubes between endogenous MURF2 proteins and ectopic LC3 and p62 proteins, we revealed that MURF2 proteins could be associated to various types of complexes engaged at various times of the autophagic process. In myoblasts, MURF2A associated with MURF2B forms Baf-sensitive and Baf-insensitive complexes with LC3 and p62. Baf-sensitive complexes containing MURF2, p62 and LC3 must be resident of autophagosome/autolysosome compartments engaged in the basal autophagic activity of myoblasts. In myoblasts, we detected more MURF2 proteins precipitated with LC3 in the absence of Baf than in its presence. Thus, MURF2A and MURF2B must link LC3 but not p62 in Baf-insensitive complexes that could localise in the phagophores. P62 can decorate various types of structures among which the autophagosome [10] and the sequestosome, defined as a cytoplasmic punctate structure without membrane confinements [9]. Recently, p62 phosphorylation was shown to regulate the recruitment of proteins to the sequestosome as the first step of their degradation by autophagy [36]. Therefore, Baf-insensitive complexes could also reflect instable MURF2/p62/LC3 complexes in the sequestosome. Although MURF2A and MURF2B do not seem related to aggresome formation we cannot exclude that they could be engaged in Baf-insensitive complexes not involved in autophagy. At the beginning of differentiation, in good agreement with activation of autophagy, we detected Baf-sensitive complexes containing MURF2, p62 and LC3. Since C2C12 differentiation is induced by serum withdrawal, we cannot determine if activation of autophagy results from serum depletion or from the differentiation process. However, since autophagy is involved at early stages of mouse development, during fasting and upon amino acid depletion or limitation of growth factors [1,37,38,39], our results must correlate with physiological processes occurring in skeletal muscles. During muscle activity, UPS plays a crucial role in both muscle adaptation and muscle atrophy [40]. To shed light on the role of both MURF2 isoforms in UPS activation, we monitored the activity of the 20S proteasome in the C2C12, RNAi MURF2A and RNAi MURF2B cells. In RNAi MURF2B myoblasts and myotubes that express high level of MURF2A, we established that the 20S proteasome activity was stimulated. Thus UPS activation could depend on MURF2A expression. During the late stage of C2C12 differentiation the increase of MURF2A protein and the decrease of polyubiquitinated proteins could result from UPS activation. Moreover, in skeletal muscle, UPS is mainly responsible for sarcomeric protein degradation and is activated during the late stages of differentiation [13,41]. However we cannot rule out that other MURF proteins could also compensate MURF2 activity and participate in the UPS activation observed in RNAi MURF2B cells. In skeletal muscle cells, MURF proteins display time-dependent expression and muscle type specificity [16]. MURF2A is expressed mainly during embryonic stages in slow muscle fibers when MURF1 and MURF3 increase after birth, MURF1 in fast fibers and MURF3 in slow and fast fibers. In cardiomyocytes, MURF2A loss is compensated by MURF3 but not MURF1 [20]. During differentiation of C2C12 cells, MURF2B protein is expressed first (Figure 1) then MURF2A, MURF1 and MURF3 appear later (Figure 1 [16]). Previous observations [16] indicated that in C2C12 cells, ablation of MURF2A by siRNA disrupts early stages of sarcomere assembly and that cell differentiation occurs later possibly due to the loss of siRNA or to the compensatory effect of MURF3. When we compared the expression of MURF1 in our MURF2 RNAi cells, we could not see differences in MURF1 protein expression between C2C12 and RNAi MURF2B cells. This suggesting that MURF1 does not compensate the loss of MURF2B and should not be directly involved in UPS activation observed in RNAi MURF2B cells (Figure S5A). Analysis of MURF3 has to be performed to determine whether it could activate UPS in the absence of MURF2B. Although autophagy can somehow compensate UPS inhibition, each degradative pathway is engaged in specific protein degradations, depending on growth conditions and cell types [42]. In agreement with these data, although RNAi MURF2B cells compensated autophagy by UPS, these cells were unable to fuse and could not form normal fibers expressing stable MTs revealed by an increase of Glu-Tubulin (Figure S5B). Since both RNAi MURF2A and RNAi MURF2B cells display impaired autophagic activity and have distinct differentiation behavior, each MURF2 protein could be engaged by their E3 ligase activity in specific UPS degradation of proteins needed or not for skeletal myogenic differentiation. Based on our results, we propose a model in which upon differentiation and/or autophagic stimulation, MURF2A could first heterodimerize with MURF2B linked to LC3 and then interact with p62 and NBR1. Therefore, due to MURF2 heterodimerization, NBR1, p62 and LC3 could be brought in close proximity to each other leading to the formation of the autophagosome. In myotubes, MURF2A decorated MTs [17] that are implicated in UPS and autophagic degradation. Indeed, upon UPS inhibition, MTs regulate the accumulation of protein aggregates at a storage compartment named the aggresome [24]. In addition, LC3 stabilizes MTs [43] and stable MTs [44] are needed for the fusion of autophagosomes to lysosomes [45]. Since MURF2A regulates the dynamics of MTs [17,18] and interacts with MURF2B, the MURF2 isoforms could also mediate the traffic of proteins or vesicles along stable MTs. MURF2A interacts with p62 (this study and [19]) a protein critical for the formation and degradation of ubiquitinated aggregates by autophagy [9]. P62 is itself degraded by autophagy [10] and also interacts with UPS [46]. Therefore, p62 acts as a general mediator in the formation and degradation of protein inclusions [47]. In muscles, upon abnormal autophagy or UPS activity, accumulation of ubiquitinated proteins may cause profound damage, as observed in various myopathies [48,49]. Thus, p62 must be tightly regulated by autophagy and UPS to maintain normal muscle functions. In myotubes that display low amounts of MURF2B, uncomplexed MURF2A could interact with p62 leading to p62 degradation by UPS and therefore inhibit autophagy. Since excess of p62 can delay UPS degradation of ubiquitinated proteins [50], regulation of the p62 homeostatic level by MURF2A could also control UPS flux. Indeed, using MURF2 RNAi cells we demonstrate that MURF2A is implicated in p62 degradation and UPS activation. These results could also explain why MURF2 knockout mice [51,52] display normal phenotype under unstressed life conditions. Additional studies should evaluate the role of the ubiquitin degradative pathways in the muscles of knockout mice after appropriate stresses activating autophagy and UPS.

In conclusion, this study indicates for the first time that in skeletal muscle cells the ratio of MURF2A and MURF2B isoforms can influence activation of autophagy or UPS protein degradation. Given the essential roles of autophagy and of UPS degradation for muscle protein homeostasy, it will be of interest to assess how defects in MURF2 protein expression or activity could correlate with myopathies.

## Materials and Methods

### Plasmids

Constructs were made by subcloning or with site-directed mutagenesis using the vectors: pEGFP and pmCyFP (Clontech), pmCherry (gift from R. Tsien) and pCMV-GST (BioTechniques 1997, *23*, 794-800). MURF2 constructs were made from pCMV5/T7-MURF2A wt and pCMV5/T7-MURF2B wt (Human proteins, EMBL database: accession numbers AJ243489 for MURF2A and AJ431704 for MURF2B, gifts from M. Gautel) to obtain: pEGFP-MURF2A wt, pEGFP-MURF2B wt, pmCherry-MURF2A wt, pmCherry-MURF2B wt and pmCyFP-MURF2B wt. Mutation of cysteine 29 and 78 to alanine yielded the MURF2 Ubiquitin dead constructs used to obtain pEGFP-MURF2A Ubi, pEGFP-MURF2B Ubi, pmCherry-MURF2A Ubi and pmCherry-MURF2B Ubi vectors. C-terminus deletion of the wt pGFP-MURF2B provided the pEGFP-MURF2Δ encompassing aa 1 to 497 of human MURF2A wt protein. Cloning of aa 505 to 537 of MURF2B and aa 505 to 545 of MURF2A into the pCMV-GST vector produced respectively the pCMV-GST-MURF2B Cter and pCMV-GST-MURF2A Cter constructs. The p62 sequence from pGST-p62 [53], subcloned into pEGFP-C1, yielded the pEGFP-p62 vector. The mCherry-p62 construct was made by exchanging the GFP gene of pEGFP-p62 with the mCherry gene of the pmCherry-C3 vector. The following vectors were described previously: pCMV5/T7-MURF1 wt and pCMV5/T7-MURF3 wt [54]. pEGFP-LC3B [55]. pDest-mCherry-NBR1 [11]. The pBABE-puro mCherry-EGFP-LC3B vector was purchased from Addgene. The pEGFP-vimentin vector was a gift from Dr R. Goldman. All constructs were verified by DNA sequencing. Oligonucleotides for cloning and DNA sequencing were obtained from Eurogentec, Sigma-Aldrich, Inc and from the EMBL facilities.

### Antibodies and reagents

Rabbit polyclonal antibodies were raised against the C-terminus of human MURF2A (aa 497-544), the C-terminus of human MURF2B (aa 504-536) and against the human MURF2 domain common to both isoforms (aa 264-435). Antibodies were prepared and purified against recombinant proteins according to standard protocols. Rabbit anti-GAPDH, rabbit anti-p62/SQSTM1, rabbit anti-LC3, rabbit anti-mono and poly Ubiquitin, mouse anti-Troponin T (clone JLT-12), mouse anti-Myogenin (clone F12B) and anti-mouse and anti-rabbit conjugated to HRP were from Sigma-Aldrich, Inc. Mouse anti-poly ubiquitin (clone FK1) was from MBL. Mouse anti-T7 tag was from Novagen, mouse anti-GFP (clones 7.1 and 13.1) from Roche and rabbit anti-GFP from Clontech. Mouse anti-GST (26H1) was from Cell Signaling Technology, mouse anti-20S proteasome subunit α 5 (clone MCP196) was from Upstate. Mouse anti-MURF1 was from Abcam. Rabbit anti Glu-Tubulin was from CHEMICON international. Goat anti-mouse or anti-rabbit secondary antibodies conjugated with various Alexa Fluor were from Molecular Probes. Goat anti-mouse antibody conjugated to Cy5 was from Jackson Immuno Research Laboratories, Inc. Bafilomycin A1, Z-Leu-Leu-Leu-Al (MG132), Dapi, Ponceau, Succinyl-leucine-leucine-valine-tyrosine-7 amido-4-methyl-coumarin and cell lytic buffer were from Sigma-Aldrich, Inc.

### ShRNA sequences

Using the pSingle-tTS-shRNA vector from Clontech vectors generating shRNA directed against mouse MURF2 proteins were constructed. ShRNA sequences were chosen according to the predicted mouse sequence (NCBI database) in comparison with the human proteins. pSingle-tTS-sh MURF2A in C2C12 yielded the RNAi MURF2A cell line. Sequences 5' TCGAGGACAGTCTGCAGCCTTGGGTTCAAGAGACCCAAGGCTGCA GACTGTCTTTTTTACGCGTA 3' and 5’ AGCTTACGCGTAAAAAAGACAGTCTGCAGC CTTGGGTCTCTTGAACCCAAGGCTGCAGACTGTCC 3' were selected to generate shRNA against the sequence GACAGTCTGCAGCCTTGGG. This sequence matches the mouse sequence located at the C-terminus of the MURF2A cDNA. pSingle-tTS-sh MURF2 in C2C12 yielded the RNAi MURF2 cell line. Sequences 5' TCGAGGATATCTACAAGCAGGAATTTCAAGAGAATTCCTGCTTGTA GATATCTTTTTTACGCGTA 3' and 5' AGCTTACGCGTAAAAAAGATATCTACAAGC AGGAATTCTCTTGAAATTCCTGCTTGTAGATATCC 3' were selected to generate shRNA against the sequence GATATCTACAAGCAGGAAT. This sequence matches the mouse sequence located in the N-terminus region of MURF2A and MURF2B cDNA. pSingle-tTS-sh MURF2B in C2C12 yielded the RNAi MURF2B cell line. Sequence 5' GATCCCCCATCTGGAGTCAGATTCAGTTCAAGAGACTGAATCTGACT CCAGATGTTTTTGGAAA 3' and 5' AGCTTTTCCAAAAACATCTGGAGTCAGATTCAG TCTCTTGAACTGAATCTGACTCCAGATGGGG 3' were selected to generate shRNA against the sequence CATCTGGAGTCAGATTCAG. This sequence matches a mouse and human sequence located at the C-terminus of the MURF2B cDNA.

### Cell lines, cell culture and transfection

To establish the MURF2 RNAi cell lines, C2C12 cells [56] were transfected with specific MURF2 pSingle-tTS-shRNA vectors and selected in 1000 µg/ml of geneticin (G418). Stable cell lines expressing the mCherry-GFP-LC3 construct were obtained by G418 selection after transfection of C2C12 and previously established MURF2 RNAi cells. All the cells were cultured in Dulbecco’s modified Eagle’s medium (DMEM) supplemented with 4.5 g glucose, 2 mM glutamine, 100 µg/ml penicillin, 100 units/ml streptomycin, 100 µg/ml gentamycin and 10% fetal calf serum (FCS). For differentiation, fetal calf serum was replaced by 1% horse serum (HS). When needed, cells were cultivated with 2 µg/ml doxycycline for 3 days prior to experiments, with MG132 (10 µM for 12 h, 50 µM for 6 h) and with 200 nM Bafilomycin A1. Cells were grown at 37°C in 5% CO2. For starvation assays, cells were incubated in Hanks’ balanced salt solution (HBSS) with 0.1% bovine serum albumin for 45 min. Transfections were performed using 1-5 µg/ml DNA and jetPEI reagent for 12 h (Polyplus transfection TM) according to the manufacturer’s instructions.

### Immunoprecipitation and immunoblotting

All RNAi and control cells were treated with doxycycline during 3 days before any type of experiment. Cells were lysed in RIPA extraction buffer (1% Triton X100, 0.5% sodium deoxycholate, 50 mM Tris pH 7.5, 150 mM NaCl, 5 mM EDTA, 5 mM EGTA) with proteinase and phophatase inhibitors (Roche Complete EDTA-free protease inhibitor cocktail, 50 mM NAF, 1 mM NaVO4 and 20 mM N ethylmaleimide). After sonication, extracts were quantified using the Pierce BCA Protein Assay Kit according to the manufacturer’s instructions. For immunoprecipitation, 300 to 400 µg of lysate were incubated overnight at 4°C with 2 µg of antibodies and magnetic beads (Bio-Adembeads pAG or GFP-Trap_M Chromotek) according to manufacturer’s instructions. For immunoblotting, 20 µg of lysate was run on NuPAGE novex 4-12% BisTris polyacrylamide gels (Invitrogen) and transferred to PROTRAN nitrocellulose membranes (Whatman). Membranes blocked and incubated with antibodies following standard procedures were visualized with Immobilon chemiluminescence reagents (Millipore) using Image Reader LAS-400 (FUJIFILMS). Protein quantification in Western blots was performed using ImageJ (National Institutes of Health, Bethesda, MD).

### Proteasome activity

The chymotrypsin-like activity of the proteasome was measured by monitoring the cleavage of the Succinyl-leucine-leucine-valine-tyrosine-7amido-4-methyl-coumarin. MG132 was used as a negative control. Protein extracts were obtained by lysing the cells with cell lytic buffer. Equal amounts of protein measured by the Bradford assay (BioRad) were incubated for 30 min with assay buffer and substrate at 37°C in the dark. The amount of 7-amino-4 methylcoumarin (AMC) was assessed in a Flex Station III.

### Immunofluorescence and image capture

Cells on coverslips rinsed with 37°C BRB80 buffer (400 mM pipes, pH 6.8-6.9, 5 mM MgCl2 and 5 mM EGTA) were fixed in a mixture of 3% paraformaldehyde, 0.1% glutaraldehyde and 0.1% Triton X-100 in BRB80 for 10 min at 37°C. After fixation, coverslips were quenched in 50 mM NH4Cl in PBS for 15 min. For immunofluorescence, coverslips were blocked in PBS containing 10% FCS and 0.1% saponin for 1 h then incubated with primary antibodies diluted in 10% FCS for 1 h. Following washes in PBS with 0.1% saponin, fluorescent secondary antibodies were applied for 1 h. Coverslips were mounted in Dako fluorescence mounting medium (DakoCytomation) and observed with an LSM 700 confocal microscope (Zeiss) equipped with a 60X objective. Images were mounted using Adobe Photoshop 7.0.

## Supporting Information

Figure S1 **Analysis of MURF2 antibody specificity.** (A) The specificity of the purified Cter-2A and Alter-2B antibodies was established by the recognition of their specific epitope tagged with GST and expressed in *E. coli*. Untransformed *E. coli* extract was used as negative control and the expressed protein revealed by the GST antibody. (B) Immunoblots performed with lysates obtained from undifferentiated C2C12 cells and after various days of differentiation (Diff.). Western blots were probed with the purified Cter-2A antibody incubated or not with MURF2A protein purified from *E. coli* (Cter-2A depletion). Ponceau red staining indicates loaded proteins. (C) The same C2C12 cell extracts were used to perform immunoblots. Purified Alter-2B antibody incubated or not with purified *E. coli* MURF2B protein (Alter-2B depletion) were used. (D) Lysates from Hela cells transfected with T7 tagged-MURF1, -MURF2B or -MURF3 were used for Western blot analyses to test All-MURF2 antibody specificity. Exogenous proteins were revealed by specific T7 antibody (T7). Purified All-MURF2 antibody incubated or not with purified *E. coli* MURF2A protein (All-MURF2 depletion) were used. Asterisks indicate unspecific signals.
(TIF)

Figure S2 **Location of MURF2A and MURF2B impaired in their ubiquitin ligase activity (MURF2A Ubi and MURF2B Ubi) and resident proteins of autophagic vesicles.** Plasmids expressing the mCherry (red) and GFP (green) tagged proteins mentioned were used. Various combinations of plasmids were transfected in C2C12 myoblasts and observed directly by confocal microscopy. Scale Bars: 10 µm.
(TIF)

Figure S3 **Analysis of the vimentin network of cells overexpressing MURF2 isoforms.** As a control, single transfection of C2C12 cells was performed with GFP-vimentin. Double transfections were performed using GFP-vimentin and mCherry-MURF2A or mCherry-MURF2B or mCherry-MURF2A Ubi or mCherry-MURF2B Ubi. The mCherry (red) and GFP (green) fluorescences were directly observed by confocal microscopy. Scale Bar: 10 µm.
(TIF)

Figure S4 **Analysis of MURF2 RNAi cell lines.** (A) Immunoblots were performed with C2C12, RNAi MURF2A, RNAi MURF2 and RNAi MURF2B lysates obtained from undifferentiated cells, and with cells differentiated for various hours as indicated. Cells were cultivated in absence (-) or in presence of 2 µg/ml doxycycline (Dox). Dox was added 24h in the growing cell medium (Diff. 0h) and during 24h, 48h and 72h in the differentiating cell medium. RNAi MURF2B cell analysis was performed by comparing lysates from undifferentiated C2C12 cells with lysates from RNAi MURF2B myoblasts (Diff. 0h) and after differentiation (Diff. 24h and 48h). Western blots were probed with the indicated antibodies. GAPDH antibody was used as control of loaded proteins. (B) Immunofluorescence experiments were performed with undifferentiated (Diff. 0h) C2C12 and MURF2 RNAi cells kept 3 days with Dox and with the Alter-2B antibody (green). (C) Immunofluorescence experiments were performed with C2C12 and MURF2 RNAi cells kept 3 days with Dox, then differentiated for 48h in presence of Dox. Cells were labeled with the Cter-2A antibody (green). Nuclei were counterstained with Dapi (blue) and cells observed by confocal microscopy. Scale bars: 10 µm.
(TIF)

Figure S5 **A) Analysis of MURF2A and MURF1 in RNAi cell lines.** Immunoblots were performed with C2C12, RNAi MURF2A and RNAi MURF2B lysates obtained from undifferentiated cells (Diff. 0h) and after 24h of differentiation. Western blots were probed with the indicated antibodies. GAPDH antibody was used as control of loaded proteins. B) Analysis of the differentiation of C2C12, RNAi MURF2A and RNAi MURF2B cells. Same number of the different cells were seed in growing medium for 24h then induced to differentiate for 72h in 1% HS. Cell were visualized by phase contrast and immunofluorescence using an anti-Glu tubulin antibody in order to visualize stable MTs. Scale Bar : 50 µm.
(TIF)

## References

[1] Sandri M (2010) Autophagy in skeletal muscle. FEBS Lett 584: 1411-1416. doi:10.1016/j.febslet.2010.01.056. PubMed: 20132819. 20132819

[2] Kachaeva EV, Shenkman BS (2012) Various jobs of proteolytic enzymes in skeletal muscle during unloading: facts and speculations. J Biomed Biotechnol, 2012: 2012: ID 493618. PubMed: 22496611 10.1155/2012/493618 PMC3303694 22496611

[3] Portbury AL, Willis MS, Patterson C (2011) Tearin’up my heart: proteolysis in the cardiac sarcomere. J Biol Chem 286: 9929-9934. doi:10.1074/jbc.R110.170571. PubMed: 21257759. 21257759 PMC3060546

[4] Mammucari C, Milan G, Romanello V, Masiero E, Rudolf R et al. (2007) FoxO3 controls autophagy in skeletal muscle in vivo. Cell Metab 6: 458-471. doi:10.1016/j.cmet.2007.11.001. PubMed: 18054315. 18054315

[5] Xie Z, Klionsky DJ (2007) Autophagosome formation: core machinery and adaptations. Nat Cell Biol 9: 1102-1109. doi:10.1038/ncb1007-1102. PubMed: 17909521. 17909521

[6] Mizushima N, Yoshimori T, Ohsumi Y (2011) The role of Atg proteins in autophagosome formation. Annu Rev Cell Dev Biol 27: 107-132. doi:10.1146/annurev-cellbio-092910-154005. PubMed: 21801009. 21801009

[7] Kabeya Y, Mizushima N, Ueno T, Yamamoto A, Kirisako T et al. (2000) LC3, a mammalian homologue of yeast Apg8p, is localized in autophagosome membranes after processing. EMBO J 19: 5720-5728. doi:10.1093/emboj/19.21.5720. PubMed: 11060023. 11060023 PMC305793

[8] Mizushima N, Yoshimori T, Levine B (2010) Methods in mammalian autophagy research. Cell 140: 313-326. doi:10.1016/j.cell.2010.01.028. PubMed: 20144757. 20144757 PMC2852113

[9] Bjørkøy G, Lamark T, Brech A, Outzen H, Perander M et al. (2005) P62/SQSTM1 forms protein aggregates degraded by autophagy and has a protective effect on huntingtin-induced cell death. J Cell Biol 171: 603-614. doi:10.1083/jcb.200507002. PubMed: 16286508. 16286508 PMC2171557

[10] Pankiv S, Hoyvarde Clausen T, Lamark T, Brech A, Bruun JA et al. (2007) P62/SQSTM1 binds directly to Atg8/LC3 to facilitate degradation of ubiquitinated protein aggregates by autophagy. J Biol Chem 282: 24131-24145. doi:10.1074/jbc.M702824200. PubMed: 17580304. 17580304

[11] Kirkin V, Lamark T, Sou YS, Bjørkøy G, Nunn JL et al. (2009) A role for NBR1 in autophagosomal degradation of ubiquitinated substrates. Mol Cell 33: 505-516. doi:10.1016/j.molcel.2009.01.020. PubMed: 19250911. 19250911

[12] Coux O, Tanaka K, Goldberg AL (1996) Strucure and function of the 20S and 26S proteasomes. Annu Rev Biochem 65: 801-847. doi:10.1146/annurev.bi.65.070196.004101. PubMed: 8811196. 8811196

[13] Solomon V, Goldberg AL (1996) Importance of the ATP-ubiquitin-proteasome pathway in the degradation of soluble and myofibrillar proteins in rabbit muscle extracts. J Biol Chem 271: 26690-26697. doi:10.1074/jbc.271.43.26690. PubMed: 8900146. 8900146

[14] Taillandier D, Combaret L, Pouch MN, Samuels SE, Béchet D et al. (2004) The role of ubiquitin-proteasome-dependent proteolysis in the remodelling of skeletal muscle. Proc Nutr Soc 63: 310-321. PubMed: 15294055. 10.1079/PAR2004358 15294055

[15] Centner T, Yano J, Kimura E, McElhinny AS, Pelin K et al. (2001) Identification of muscle specific ring finger proteins as potential regulators of the Titin kinase domain. J Mol Biol 306: 717-726. doi:10.1006/jmbi.2001.4448. PubMed: 11243782. 11243782

[16] Perera S, Mankoo B, Gautel M (2012) Developmental regulation of MURF E3 ubiquitin ligases in skeletal muscle. J Muscle Res Cell Motil 33: 107–122. doi:10.1007/s10974-012-9288-7. PubMed: 22426552. 22426552 PMC3353113

[17] Pizon V, Iakovenko A, van der Ven PF, Kelly R, Fatu C et al. (2002) Transient association of titin and myosin with microtubules in nascent myofibrils directed by the MURF2 RING-finger protein. J Cell Sci 115: 4469-4482. doi:10.1242/jcs.00131. PubMed: 12414993. 12414993

[18] McElhinny AS, Perry CN, Witt CC, Labeit S, Gregorio CC (2004) Muscle-specific RING finger-2 (MURF-2) is important for microtubule, intermediate filament and sarcomeric M-line maintenance in striated muscle development. J Cell Sci 117: 3175-3188. doi:10.1242/jcs.01158. PubMed: 15199100. 15199100

[19] Lange S, Xiang F, Yakovenko A, Vihola A, Hackman P et al. (2005) The kinase domain of Titin controls muscle gene expression and protein turnover. Science 308: 1599-1603. doi:10.1126/science.1110463. PubMed: 15802564. 15802564

[20] Perera S, Holt MR, Mankoo BS, Gautel M (2011) Developmental regulation of MURF ubiquitin ligases and autophagy proteins nbr1, p62/SQSTM1 and LC3 during cardiac myofibril assembly and turnover. Dev Biol 351: 46–61. doi:10.1016/j.ydbio.2010.12.024. PubMed: 21185285. 21185285 PMC3047806

[21] Borden KL (2000) Ring domains: master builders of molecular scaffolds? J Mol Biol 295: 1103-1112. doi:10.1006/jmbi.1999.3429. PubMed: 10653689. 10653689

[22] Deshaies RJ, Joazeiro CA (2009) Ring domain E3 ubiquitin ligases. Annu Rev Biochem 78: 399-434. doi:10.1146/annurev.biochem.78.101807.093809. PubMed: 19489725. 19489725

[23] Spencer JA, Eliazer S, Ilaria RL Jr, Richardson JA, Olson EN (2000) Regulation of microtubule dynamics and myogenic differentiation by MURF, a striated muscle ring-finger protein. J Cell Biol 150: 771-784. doi:10.1083/jcb.150.4.771. PubMed: 10953002. 10953002 PMC2175279

[24] Kopito RR (2000) Aggresomes, inclusion bodies and protein aggregation. Trends Cell Biol 10: 524-530. doi:10.1016/S0962-8924(00)01852-3. PubMed: 11121744. 11121744

[25] Johnston JA, Ward CL, Kopito RR (1998) Aggresomes: a cellular response to misfolded proteins. J Cell Biol 143: 1883-1898. doi:10.1083/jcb.143.7.1883. PubMed: 9864362. 9864362 PMC2175217

[26] Terje J, Lamark T (2010) Selective autophagy mediated by autophagic adapter proteins. Autophagy 7: 279-296. PubMed: 21189453. 10.4161/auto.7.3.14487 PMC3060413 21189453

[27] Itakura E, Mizushima N (2011) P62 targeting to the autophagosome formation site requires self-oligomerization but not LC3 binding. J Cell Biol 192: 17-27. doi:10.1083/jcb.201009067. PubMed: 21220506. 21220506 PMC3019556

[28] Klionsky DJ, Elazar Z, Seglen PO, Rubinsztein DC (2008) Does bafilomycin A1 block the fusion of autophagosomes with lysosomes? Autophagy 4: 849-950. PubMed: 18758232. 18758232 10.4161/auto.6845

[29] Kabeya Y, Mizushima N, Yamamoto A, Oshitani-Okamoto S, Ohsumi Y et al. (2004) LC3, GABARAP and GATE16 localize to autophagosomal membrane depending on form-II formation. J Cell Sci 117: 2805-2812. doi:10.1242/jcs.01131. PubMed: 15169837. 15169837

[30] Meiners S, Heyken D, Weller A, Ludwig A, Stangl K et al. (2003) Inhibition of proteasome activity induces concerted expression of proteasome genes and de novo formation of mammalian proteasomes. J Biol Chem 278: 21517-21525. doi:10.1074/jbc.M301032200. PubMed: 12676932. 12676932

[31] Li Y-P, Chen Y, Li A, Reid M (2003) Hydrogen peroxide stimulates ubiquitin-conjugating activity and expression of genes for specific E2 and E3 proteins in skeletal muscles myotubes. Am J Physiol Cell Physiol 285: 806-812. doi:10.1152/ajpcell.00129.2003. 12773310

[32] Kuusisto E, Suuronen I, Salminen A (2001) Ubiquitin-binding protein p62 expression is induced during apoptosis and proteasome inhibition in neuronal cells. Biochem Biophys Res Commun 280: 223-228. doi:10.1006/bbrc.2000.4107. PubMed: 11162503. 11162503

[33] Witt SH, Granzier H, Witt CC, Labeit S (2005) MURF-1 and MURF-2 target specific subset of myofibrillar proteins redundantly: towards understanding MURF- dependent muscle ubiquitination. J Mol Biol 350: 713-722. doi:10.1016/j.jmb.2005.05.021. PubMed: 15967462. 15967462

[34] Gregorio CC, Perry CN, McElhinny AS (2005) Functional properties of the titin/connectin-associated proteins, the muscle-specific RING finger proteins (MURFs), in striated muscle. J Muscle Res Cell Motil 26: 389-400. PubMed: 16477476. 16477476 10.1007/s10974-005-9021-x

[35] Musa H, Meek S, Gautel M, Peddie D, Smith AJ et al. (2006) Targeted homozygous deletion of M-band titin in cardiomyocytes prevents sarcomere formation. J Cell Sci 119: 4322-4331. doi:10.1242/jcs.03198. PubMed: 17038546. 17038546

[36] Matsumoto G, Wada K, Okuno M, Kurosawa M, Nukina N (2011) Serine 403 phosphorylation of p62/SQSTM1 regulates selective autophagic clearance of ubiquitinated proteins. Cell 44: 279-291. PubMed: 22017874. 10.1016/j.molcel.2011.07.039 22017874

[37] Yue Z, Yang C, Levine A, Heintz N (2003) Beclin 1, an autophagy gene essential for early embryonic development, is a haploinsufficient tumor suppressor. Proc Natl Acad Sci U S A 100: 15077-15082. doi:10.1073/pnas.2436255100. PubMed: 14657337. 14657337 PMC299911

[38] Tsukamoto S, Kuma A, Murakami M, Kishi C, Yamamoto A et al. (2008) Autophagy is essential for preimplantation development of mouse embryos. Science 321: 117-120. doi:10.1126/science.1154822. PubMed: 18599786. 18599786

[39] Rabinowitz JD, White E (2010) Autophagy and metabolim. Science 330: 1344-1348. doi:10.1126/science.1193497. PubMed: 21127245. 21127245 PMC3010857

[40] Bodine SC, Latres E, Baumhueter S, Lai VK, Nunez L et al. (2001) Identification of ubiquitin ligases required for skeletal muscle atrophy. Science 294: 1704-1708. doi:10.1126/science.1065874. PubMed: 11679633. 11679633

[41] Purintrapiban J, Wang MC, Forsberg NE (2003) Degradation of sarcomeric and cytoskeletal proteins in culture skeletal muscle cells. Comp Biochem Physiol B Biochem Mol Biol 136: 393-401. doi:10.1016/S1096-4959(03)00201-X. PubMed: 14602148. 14602148

[42] Fuertes G, Martín De Llano J, Villarroya A, Rivett J, Knecht E (2003) Changes in the proteolytic activities of proteasomes and lysosomes in human fibroblasts produced by serum withdrawal, amino-acid deprivation and confluent conditions. Biochem J 375: 75-86. doi:10.1042/BJ20030282. PubMed: 12841850. 12841850 PMC1223664

[43] Faller EM, Villeneuve TS, Brown DL (2009) Map1a associated light chain3 increases microtubule stability by suppressing microtubule dynamics. Mol Cell Neurosci 41: 85-93. doi:10.1016/j.mcn.2009.02.001. PubMed: 19233279. 19233279

[44] Gundersen GG, Khawaja S, Bulinski JC (1989) Generation of a stable, posttranslationally modified microtubule array is an early event in myogenic differentiation. J Cell Biol 109: 2275-2288. doi:10.1083/jcb.109.5.2275. PubMed: 2681230. 2681230 PMC2115884

[45] Xie R, Nguyen S, McKeehan W, Liu L (2010) Acetylated microtubules are required for fusion of autophagosomes with lysosomes. BMV. Cell Biol 11: 89-101. 10.1186/1471-2121-11-89 PMC2995476 21092184

[46] Seibenhener ML, Babu JR, Geetha T, Wong HC, Krishna NR et al. (2004) Sequestosome 1/p62 is a polyubiquitin chain binding protein involved in ubiquitin proteasome degradation. Mol Cell Biol 24: 8055-8068. doi:10.1128/MCB.24.18.8055-8068.2004. PubMed: 15340068. 15340068 PMC515032

[47] Komatsu M, Waguri S, Koike M, Sou YS, Ueno Y et al. (2007) Homeostatic levels of p62 control cytoplasmic inclusion body formation in autophagy-deficient mice. Cell 14: 1149-1163. PubMed: 18083104. 10.1016/j.cell.2007.10.035 18083104

[48] Weihl CC, Miller SE, Hanson PI, Pestronk A (2007) Transgenic expression of inclusion body myopathy associated mutant p97/VCP causes weakness and ubiquitinated protein inclusions in mice. Hum Mol Genet 16: 919-928. doi:10.1093/hmg/ddm037. PubMed: 17329348. 17329348

[49] Raben N, Hill V, Shea L, Takikita S, Baum R et al. (2008) Suppression of autophagy in skeletal muscle uncovers the accumulation of ubiquitinated proteins and their potential role in muscle damage in Pompe disease. Hum Mol Genet 17: 3897-3908. doi:10.1093/hmg/ddn292. PubMed: 18782848. 18782848 PMC2638578

[50] Korolchuck V, Mansilla A, Menzies FM, Rubinsztein DC (2009) Autophagy inhibition compromises degradation of ubiquitin-proteasome pathway substrates. Mol Cell 33: 517-527. doi:10.1016/j.molcel.2009.01.021. PubMed: 19250912. 19250912 PMC2669153

[51] Witt CC, Witt SH, Lerche S, Labeit D, Back W et al. (2008) Cooperative control of striated muscle mass and metabolism bu MURF1 and MURF2. EMBO J 27: 350-360. doi:10.1038/sj.emboj.7601952. PubMed: 18157088. 18157088 PMC2168395

[52] Willis MS, Ike C, Li L, Wang DZ, Glass DJ et al. (2007) Muscle ring finger 1, but not muscle ring finger 2, regulates cardiac hypertrophy in vivo. Circ Res 100: 456-459. doi:10.1161/01.RES.0000259559.48597.32. PubMed: 17272810. 17272810 PMC4112093

[53] Gal J, Ström A-L, Kilty R, Zhang F, Zhu H (2007) p62 accumulates and enhances aggregate formation in model systems of familial amyotrophic lateral sclerosis. J Biol Chem 282: 11068-11077. doi:10.1074/jbc.M608787200. PubMed: 17296612. 17296612

[54] Iakovenko A, Gautel M (2000) Titin-associated zinc finger proteins link titin kinase to transcriptional control. J Muscle Res Cell Motil 21: 833.

[55] Simonsen A, Birkeland HC, Gillooly DJ, Mizushima N, Kuma A et al. (2004) Alfy, a novel FYVE-domain-containing protein associated with protein granules and autophagic membranes. J Cell Sci 117: 4239-4251. doi:10.1242/jcs.01287. PubMed: 15292400. 15292400

[56] Yaffe D, Saxel O (1977) Serial passaging and differentiation of myogenic cells isolated from dystrophic mouse muscle. Nature 270: 725-772. doi:10.1038/270725a0. PubMed: 563524. 563524

