# Supplementary figures and images for: MURF2B, a Novel LC3-Binding Protein, Participates with MURF2A in the Switch between Autophagy and Ubiquitin Proteasome System during Differentiation of C2C12 Muscle Cells
Source: PLoS One. 2013 Oct 4;8(10):e76140. doi: 10.1371/journal.pone.0076140 (PMC3790703; doi:10.1371/journal.pone.0076140)

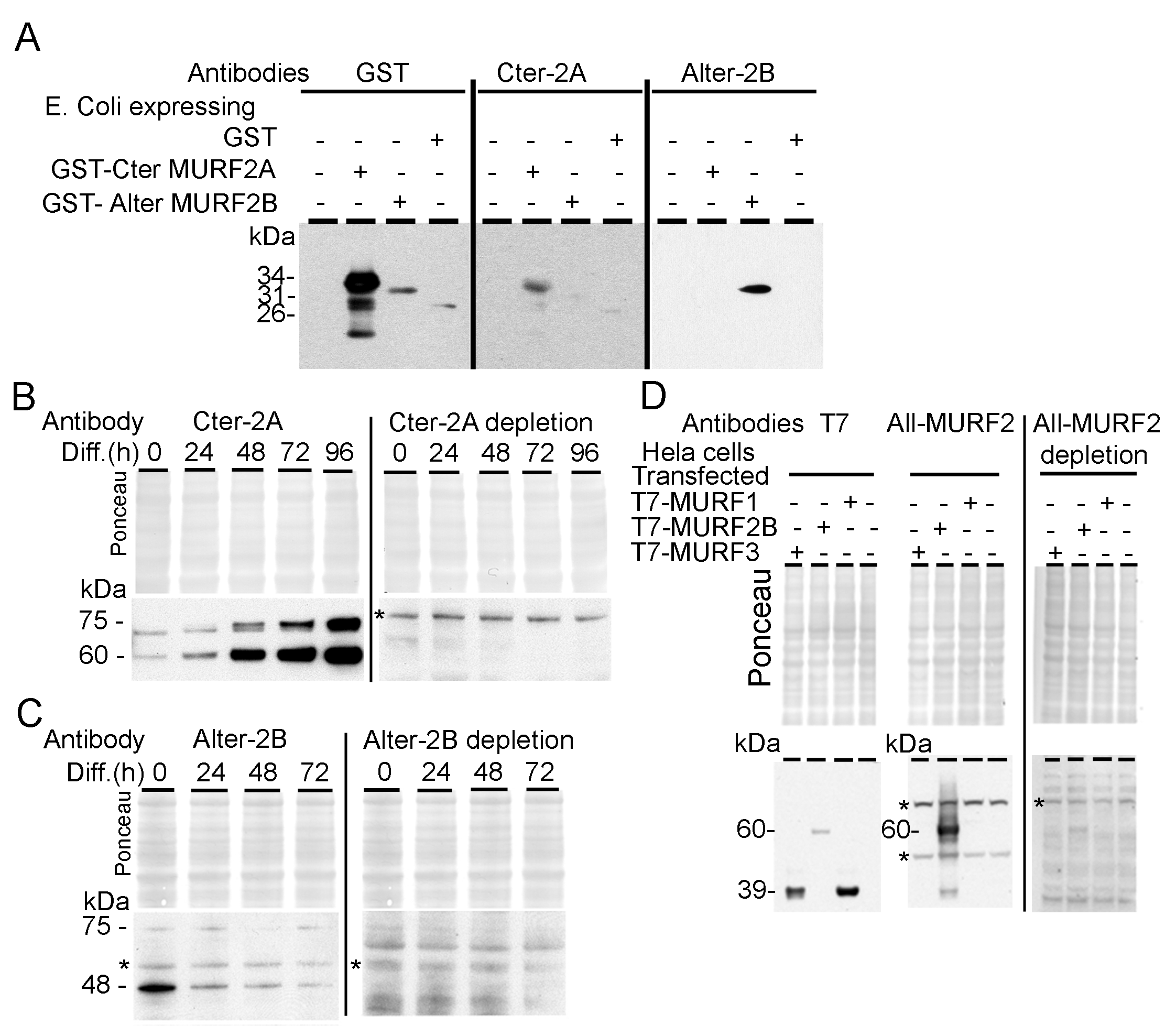

Supplement: Figure S1 — Analysis of MURF2 antibody specificity. (A) The specificity of the purified Cter-2A and Alter-2B antibodies was established by the recognition of their specific epitope tagged with GST and expressed in E. coli. Untransformed E. coli extract was used as negative control and the expressed protein revealed by the GST antibody. (B) Immunoblots performed with lysates obtained from undifferentiated C2C12 cells and after various days of differentiation (Diff.). Western blots were probed with the purified Cter-2A antibody incubated or not with MURF2A protein purified from E. coli (Cter-2A depletion). Ponceau red staining indicates loaded proteins. (C) The same C2C12 cell extracts were used to perform immunoblots. Purified Alter-2B antibody incubated or not with purified E. coli MURF2B protein (Alter-2B depletion) were used. (D) Lysates from Hela cells transfected with T7 tagged-MURF1, -MURF2B or -MURF3 were used for Western blot analyses to test All-MURF2 antibody specificity. Exogenous proteins were revealed by specific T7 antibody (T7). Purified All-MURF2 antibody incubated or not with purified E. coli MURF2A protein (All-MURF2 depletion) were used. Asterisks indicate unspecific signals. (TIF) [file pone.0076140.s001.tif]

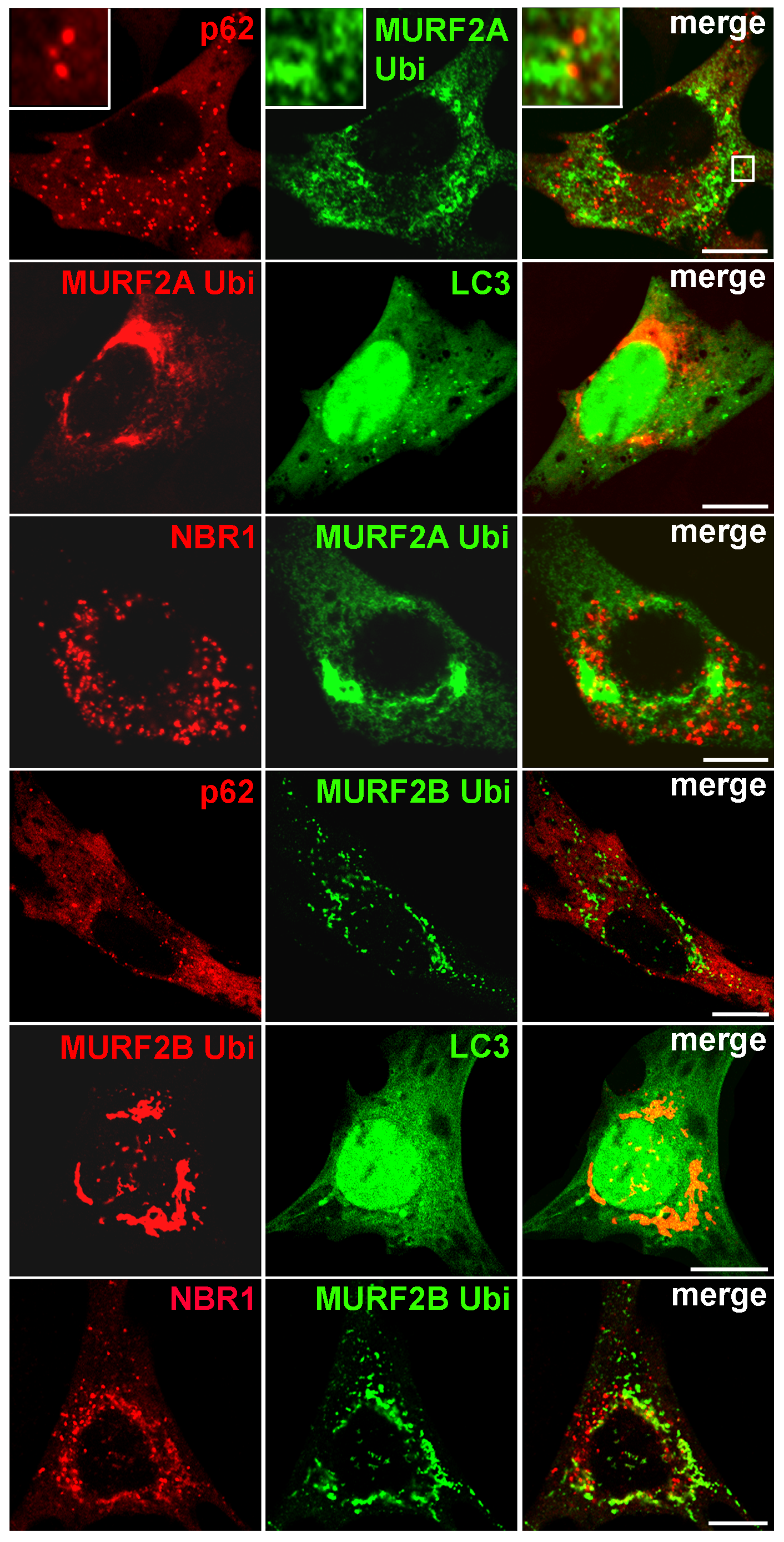

Supplement: Figure S2 — Location of MURF2A and MURF2B impaired in their ubiquitin ligase activity (MURF2A Ubi and MURF2B Ubi) and resident proteins of autophagic vesicles. Plasmids expressing the mCherry (red) and GFP (green) tagged proteins mentioned were used. Various combinations of plasmids were transfected in C2C12 myoblasts and observed directly by confocal microscopy. Scale Bars: 10 µm. (TIF) [file pone.0076140.s002.tif]

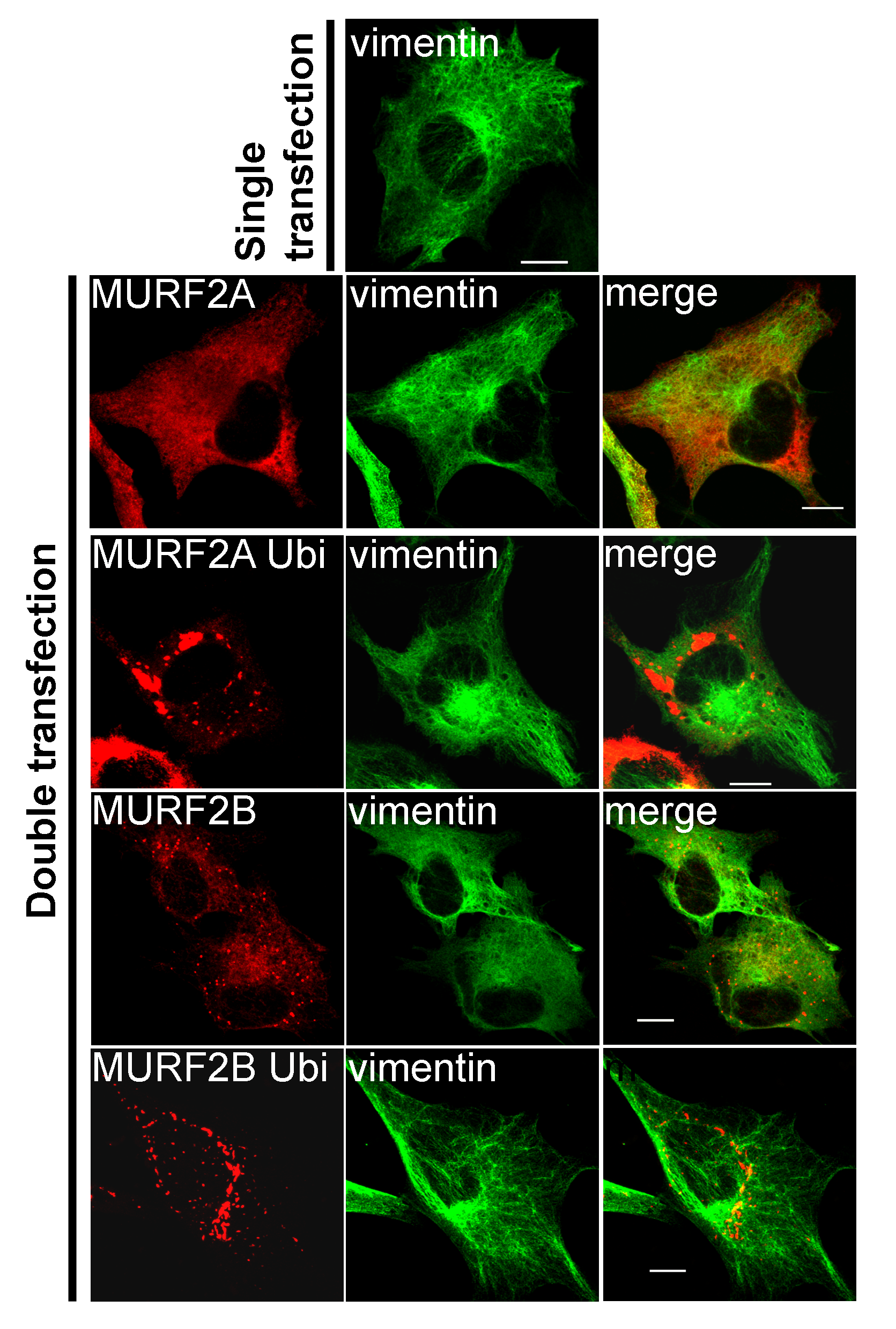

Supplement: Figure S3 — Analysis of the vimentin network of cells overexpressing MURF2 isoforms. As a control, single transfection of C2C12 cells was performed with GFP-vimentin. Double transfections were performed using GFP-vimentin and mCherry-MURF2A or mCherry-MURF2B or mCherry-MURF2A Ubi or mCherry-MURF2B Ubi. The mCherry (red) and GFP (green) fluorescences were directly observed by confocal microscopy. Scale Bar: 10 µm. (TIF) [file pone.0076140.s003.tif]

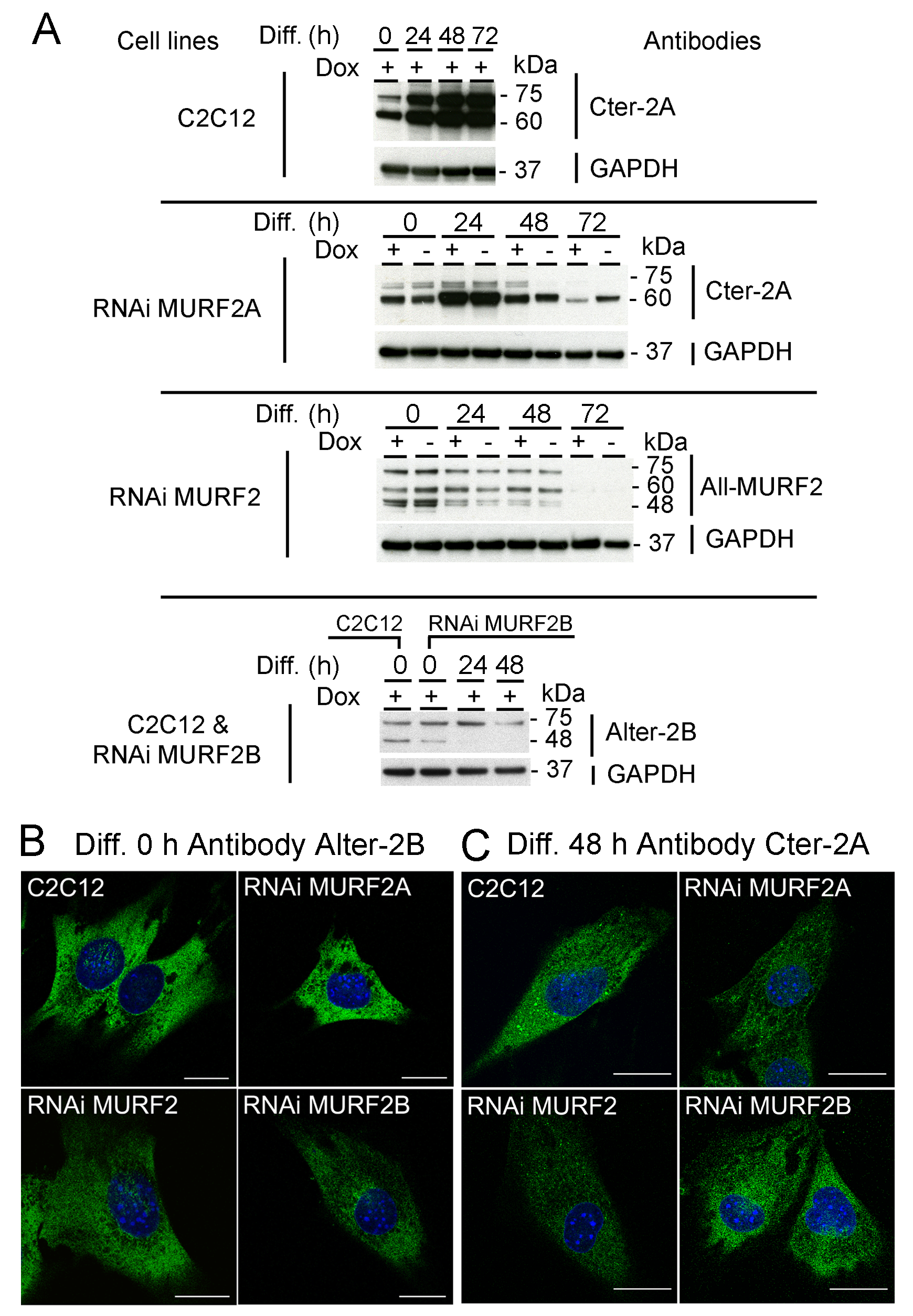

Supplement: Figure S4 — Analysis of MURF2 RNAi cell lines. (A) Immunoblots were performed with C2C12, RNAi MURF2A, RNAi MURF2 and RNAi MURF2B lysates obtained from undifferentiated cells, and with cells differentiated for various hours as indicated. Cells were cultivated in absence (-) or in presence of 2 µg/ml doxycycline (Dox). Dox was added 24h in the growing cell medium (Diff. 0h) and during 24h, 48h and 72h in the differentiating cell medium. RNAi MURF2B cell analysis was performed by comparing lysates from undifferentiated C2C12 cells with lysates from RNAi MURF2B myoblasts (Diff. 0h) and after differentiation (Diff. 24h and 48h). Western blots were probed with the indicated antibodies. GAPDH antibody was used as control of loaded proteins. (B) Immunofluorescence experiments were performed with undifferentiated (Diff. 0h) C2C12 and MURF2 RNAi cells kept 3 days with Dox and with the Alter-2B antibody (green). (C) Immunofluorescence experiments were performed with C2C12 and MURF2 RNAi cells kept 3 days with Dox, then differentiated for 48h in presence of Dox. Cells were labeled with the Cter-2A antibody (green). Nuclei were counterstained with Dapi (blue) and cells observed by confocal microscopy. Scale bars: 10 µm. (TIF) [file pone.0076140.s004.tif]

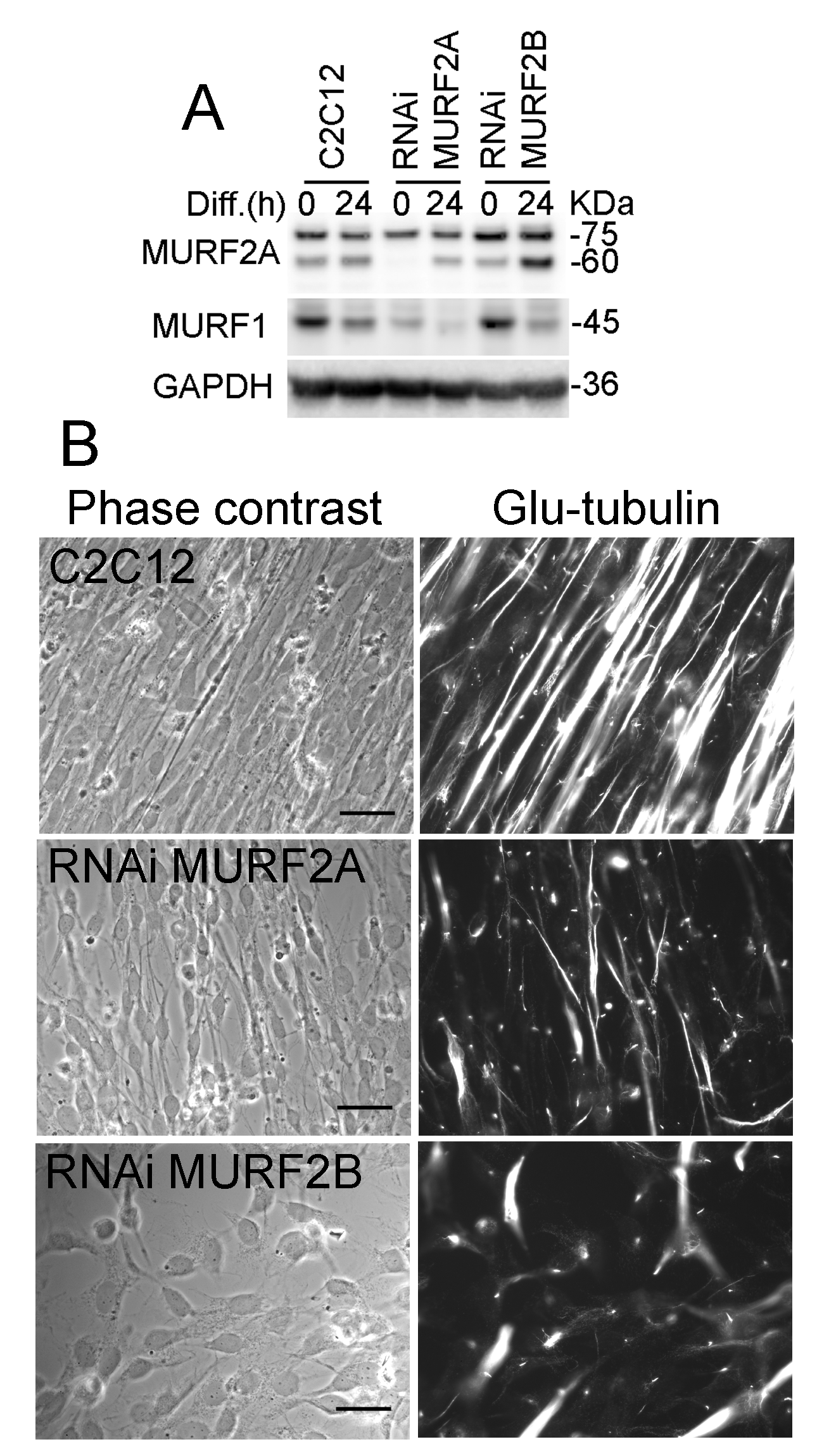

Supplement: Figure S5 — A) Analysis of MURF2A and MURF1 in RNAi cell lines. Immunoblots were performed with C2C12, RNAi MURF2A and RNAi MURF2B lysates obtained from undifferentiated cells (Diff. 0h) and after 24h of differentiation. Western blots were probed with the indicated antibodies. GAPDH antibody was used as control of loaded proteins. B) Analysis of the differentiation of C2C12, RNAi MURF2A and RNAi MURF2B cells. Same number of the different cells were seed in growing medium for 24h then induced to differentiate for 72h in 1% HS. Cell were visualized by phase contrast and immunofluorescence using an anti-Glu tubulin antibody in order to visualize stable MTs. Scale Bar : 50 µm. (TIF) [file pone.0076140.s005.tif]
